# Analyzing gaze and hand movement patterns in leader-follower interactions during a time-continuous cooperative manipulation task

**DOI:** 10.3389/fpsyg.2025.1699261

**Published:** 2026-01-06

**Authors:** Minghao Cheng, Anoushiravan Zahedi, Ricarda I. Schubotz, Florentin Wörgötter, Minija Tamosiunaite

**Affiliations:** 1 Institute Physics 3, Computational Neuroscience, Georg-August University Göttingen, Göttingen, Germany; 2 Department of Psychology, University of Münster, Münster, Germany; 3 Otto-Creutzfeldt-Center of Behavioral and Cognitive Neuroscience, University of Münster, Münster, Germany; 4 Faculty of Informatics, Vytautas Magnus University, Kaunas, Lithuania

**Keywords:** action-counteraction game, Bayesian hierarchical modeling, eye and hand coordination, eye tracking, joint action, leader-follower interaction

## Abstract

In daily life, people often interact by taking on leader and follower roles. Unlike laboratory experiments, these interactions unfold naturally and continuously. Although it is well established that gaze typically precedes object manipulation, much less is known about how gaze–hand patterns evolve in interactive settings where one person must take the other’s actions into account. Here, we examine predictive, planning-related behavior in a two-player tabletop game called “do-undo.” Participants alternated as Leader and Follower. The Leader performed simple pick-and-place actions to alter the arrangement of objects, while the Follower used other objects to restore the previous configuration. We recorded eye and hand movements, along with object trajectories, using a system that combined eye tracking with multi-camera motion capture. Touch sensors on the players’ hands provided precise timing of contacts, allowing us to segment cooperative action into well-defined temporal intervals. As expected, eye fixations consistently preceded manipulation, but clear role differences emerged. Leaders looked more often and earlier at target objects. Further, Leaders’ gaze anticipated not only their own actions but also those of the Followers. Leaders also more frequently checked the outcome of the do-undo sequence. Both roles showed gaze patterns consistent with memorization, but alternating gazes between objects and destinations were much more common in Leaders. Some patterns suggested longer-term planning beyond the immediate action. These findings reveal distinct decision-making and planning strategies in Leaders and Followers. Leader-centric interactions, highlighted by Leaders considering not only their own next moves but also their partners’ potential actions, shed light on the complex cognitive processes that underlie everyday human interaction.

## Introduction

Collaborative human-human interaction occurs frequently in our daily lives (Kourtis et al., 2013; Torok et al., 2019), for example, when we assemble furniture, fix a bike, or cook a meal together. In these everyday moments of shared action, one person often takes the lead – at least for a while – and the other follows, performing complementary actions to achieve a common goal (Noy et al., 2011). While it is extensively investigated how individuals coordinate during joint tasks (e.g., McEllin et al., 2020; Schmitz et al., 2017), less is known about the effects of being a follower versus a leader on cognitive processes and attention allocation, especially during object manipulation.

Joint actions involve both agents’ continuous acquisition of information, typically through vision, to monitor their own and the other’s actions, which is reflected in reactive gazes. Reactive gazes might also be related to other cognitive processes; for instance, gazes may be directed at empty space where an object once was, reflecting memory processes (O'Regan, 1992; Foerster, 2019). However, eye movements are expected not only to follow an action, but also often to precede it, a phenomenon referred to as anticipatory gaze (Land and Hayhoe, 2001; Riechelmann et al., 2021). Anticipatory gazes are often directed to task-relevant targets shortly before the corresponding movement — a “just-in-time” mode of anticipatory gaze (Keshava et al., 2024). Anticipatory gaze can also be directed to an action planned (e.g., moving an object from location A to B) but not yet executed or completed (Land et al., 1999; Land and Hayhoe, 2001). Furthermore, patterns of repeated fixations on the same object—when the eyes return to it multiple times—have been closely associated with the cognitive processes underlying action planning (Sullivan et al., 2021; Mennie et al., 2007; Pelz and Canosa, 2001). Clearly, overall gaze patterns are complex, as gaze can also sometimes fall on action-irrelevant objects, or objects may be grasped without a preceding fixation (Hayhoe et al., 2003) or by using peripheral vision alone (Brown et al., 2005).

Not only have actors’ fixations been studied, but also those of action observers. Similar to actors, observers make predictive looks toward the actor’s to-be-manipulated objects (Flanagan and Johansson, 2003; Gredeback and Falck-Ytter, 2015), and such predictive fixations are even more common than sustained tracking of the actor’s hand (Flanagan et al., 2013). Furthermore, when an observer has previously performed the action themselves, they tend to predict earlier (Möller et al., 2015).

Despite existing knowledge of actors’ and observers’ gaze behavior, much less is known about gaze behavior during joint manipulation actions. Together, recent findings demonstrate that gaze provides a timely method for capturing the real-time dynamics of interacting minds (Hessels et al., 2024a), illuminating how people coordinate attention and build shared understanding during joint action (Wohltjen and Wheatley, 2024). Despite providing reliable behavioral markers of leadership, followership, and the hierarchical functioning of teams, eye tracking is underused (Cheng et al., 2023), especially for investigating follower-leader interactions in joint action. In the current study, we employed anticipatory and reactive gazes to investigate how different roles (e.g., leader vs. follower) affect attentional and cognitive processes involved in joint action.

Previous studies investigating human-human interactions often rely on artificial settings, including sparsely distributed objects of exaggerated size (Huang et al., 2015), virtual reality with large screens (Andrist et al., 2017; Fuchs and Belardinelli, 2021), or specially designed robotic setups for slowing down human motion (Stolzenwald and Mayol-Cuevas, 2018). Although such settings allow improved resolution of eye fixations, they render the environment less ecologically valid. From eye tracking studies of joint actions with more complex tabletop tasks performed in realistic settings, it is evident that mutual gaze coordination (i.e., looking at the same location) is significantly reduced when participants do not communicate verbally (Hessels et al., 2023). Previous studies have shown that in such demanding tasks, participants rarely look at each other’s faces (less than 0.5% of the time), and their gaze is more strongly coupled to their own actions (Hessels et al., 2024b). However, these studies did not differentiate between individual objects, only distinguishing larger table regions, rendering their results prone to alternative interpretations.

To address these limitations, our study investigated predictive (i.e., planning-related) and reactive (i.e., information-collection-related) gaze behavior in a two-player tabletop game, focusing on the leader-follower dynamic, with the aim of enhancing both the ecological validity of the task and the precision of gaze measurement. We employed a joint tabletop manipulation task, specifically focusing on hand-object interactions without verbal communication. These interactions remain gaze-intensive because one must visually plan and attend to one’s own actions to execute them correctly, while also observing what the other is doing to plan their own action accordingly. We tracked eye movements, hand movements, and individual objects on the table to determine how eye and hand movements relate to one another across players assuming different roles. Data were recorded using a setup that combined gaze tracking with multicamera motion tracking of the participants and table configuration, along with touch sensors on the hands. This setup allowed accurate phasing of cooperative manipulation based on the moments of hand contact (*touching*) and release (*untouching*) during object manipulation.

Our overarching goal was to address whether anticipatory and reactive gaze behavior would differ when participants assume the role of a leader versus a follower in this ecologically valid game. Especially since leaders must plan actions ahead, whereas followers adjust to the leader’s actions, we were interested in the interactions between role (i.e., leader vs. follower), activity (actor vs. observer), and event (grasping an object vs. placing it at a destination). To achieve this, we analyzed the number of fixations, total fixation duration, and fixation latencies using multilevel Bayesian generalized linear modeling.

## Methods

### Subjects

We conducted experiments with a total of 60 adult, right-handed participants (39 male, 21 female; age range 19–35 years), tested in pairs. All participants had normal or corrected-to-normal vision and were informed about the purpose of the experiment before providing written informed consent. The experiment was performed in accordance with the ethical standards laid down in the 1964 Declaration of Helsinki and the relevant guidelines of the DPG. The experimental procedures were approved by the Ethics Committee of the University of Göttingen, Department of Psychology (registration no. 294).

### Experimental paradigm

For this study, we designed a paradigm consisting of an Action-Counteraction (ACA) game. The two participants assumed different roles during the ACA game: Leader and Follower. The Leader was free to execute actions, while the Follower responded to the Leader’s preceding action.

**Figure 1:**
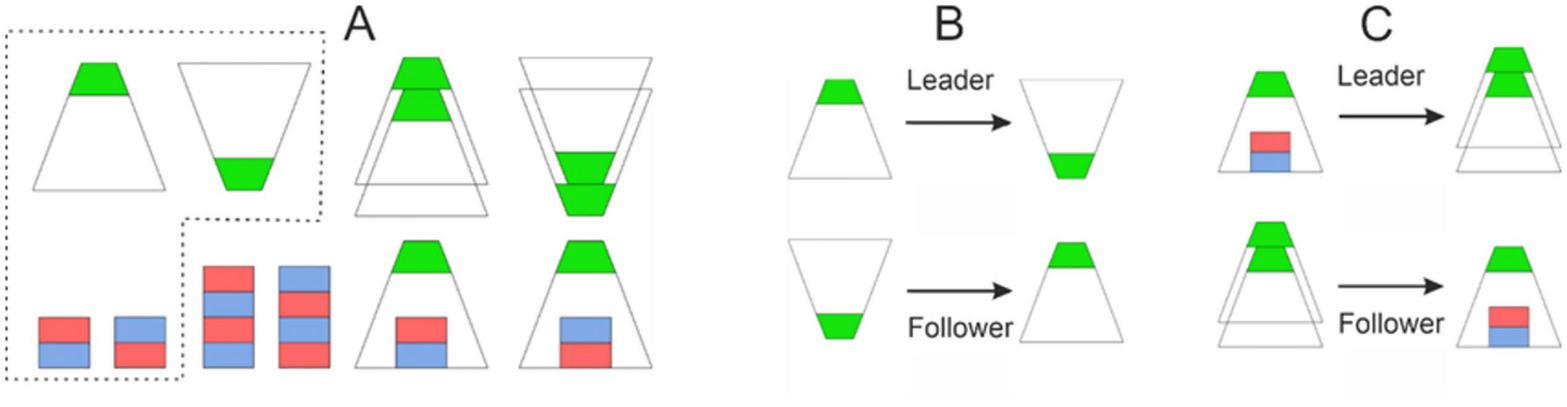
**(A)** Objects used in the ACA game and their allowed configurations. We used transparent glasses so that participants would be able to see an object if it was under a glass, while the bottom part of the glasses was painted green to allow the computer vision system to detect and recognize them. **(B)** An example of an action-counteraction pair (glass inverting) by the Leader, where the Follower has to counteract this by taking an inverted glass, turning it around somewhere else on the table. **(C)** Another example: the Leader takes an inverted glass standing over a cube and puts it over a singular inverted glass, thus forming a stack; the Follower then has to find another stack of glasses standing on the table, as well as a singular cube of the same orientation standing somewhere else on the table, to perform the counteraction.

**Figure 2:**
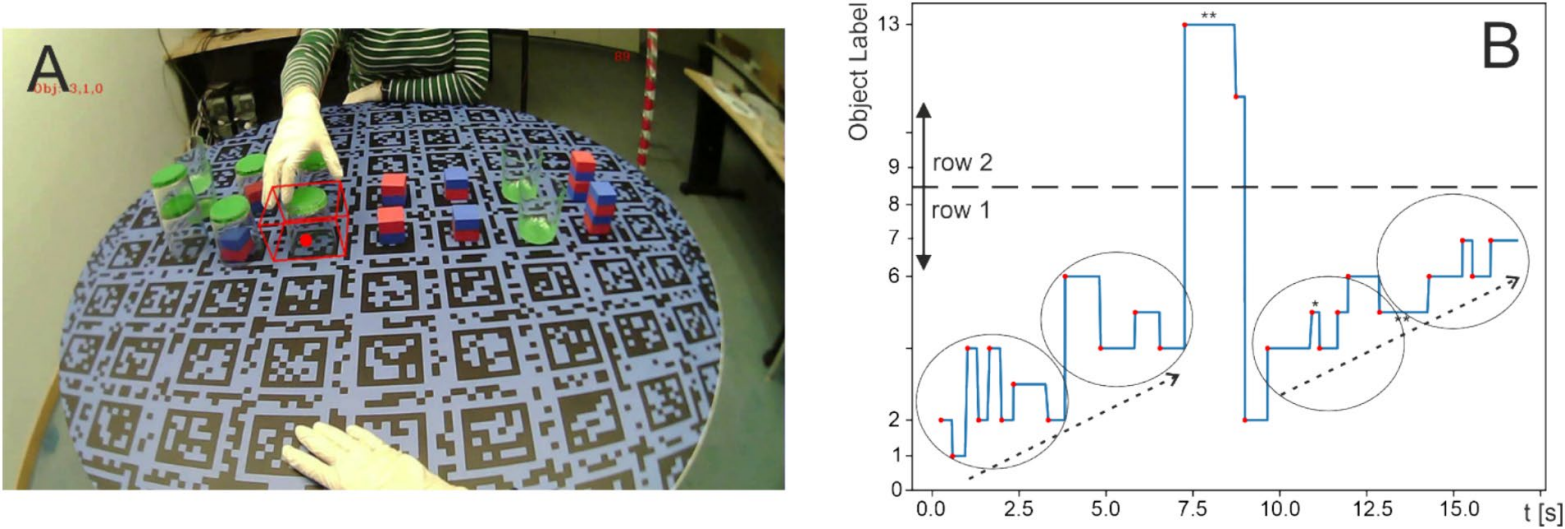
**(A)** View as seen by the eye-tracker camera, including one object bounding box and the gaze indicator (red dot) of the person in front. One can see the object configurations shown in Figure 1A distributed across 14 grid markers in the middle of the table, while on the sides of the rows, there are empty grid markers—two on the right and two on the left—where objects are also allowed to be placed during the game. This view is essentially identical to how participants see the scene. **(B)** Eye trace over time. The onsets of fixations are marked by red dots. The grid positions on which the objects are distributed on the table in A are numbered from left to right as 0-8 for the first row and 9-13 for the second row (note that positions 1, 8, 9, and 16 are empty in **A**). Accordingly, the y-axis is labeled with these numerical location markers corresponding to the positions at which fixations were directed. The dashed arrows in **B** show how the eye moves, making fixations along the first row of objects, sometimes oscillating back and forth. These local oscillations are indicated by circles. The large jump reflects the saccade to the second row. Afterwards, the eye continues along the first row.

At the start of each block, a set of glasses and cubes was placed on the table in two rows and nine columns. These glasses and cubes could have been on the table unstacked (i.e., single objects) or stacked on top of each other. In total, the set was composed of 8 cubes and 8 glasses. The initial configuration had three rules: (A) a glass can be stacked on top of a glass or a cube, but a cube could be stacked only on top of another cube; (B) when a glass is stacked on top of a cube, it can be only inverted, and (C) the initial setup should always be composed of two glasses, two inverted glasses, two cubes, two inverted cubes, and 6 stacked objects, i.e., stacked glasses, inverted stacked glasses, stacked cubes, inverted stacked cubes, a glass on top of a cube, and a glass on top of an inverted cube. Based on these rules, 14 out of 18 possible locations will be occupied in the initial setup, and four locations will remain free. Allowed object combinations are shown in Figure 1A; additionally, Figure 2A illustrates a possible setup on the table.

When the game started, the Leader performed an action, freely chosen from the allowed configurations (i.e., rules A and B). Upon completion of the Leader’s action, the Follower executed a counteraction. The counteraction had to satisfy two rules: (1) after the pair of action and counteraction, the configurations of the objects on the table must be the same as before. Therefore, after the Follower’s action, the objects on the table should have fulfilled the rule C. (2) The Follower could not manipulate the same object as the Leader. Figures 1B,C display two examples of possible action-counteraction in the game. Furthermore, an example Leader-Follower action-counteraction is shown in the Supplementary Video “action-counteraction experiment.mp4”. The setup was designed so that a counteraction is always possible for any of the allowed actions that the Leader can take. In the above defined setup, the Leader had always 14 affordances for grasping an object and 5 to 11 affordances for the destination. The Follower had 1 to 3 affordances for grasping and 1 to 5 affordances for the destination.

After each action pair, the Leader and the Follower performed the next pair, repeating this for 10 rounds. Then the two players swapped roles and performed 10 more rounds. After 20 rounds, the experimental session ended.

### Experimental procedure

Participants sat opposite each other at a round table (see Figure 2A). Each session began with the experimenter explaining the game rules and allowing participants to practice until familiar. Once both participants were ready, they put on white gloves to improve the computer vision system’s recognition of their hands. In addition, the gloves contained a microswitch under the index finger to accurately record touching events. Eye-trackers (Pupil Core eye trackers from Pupil Labs) were attached to the participants, similar to wearing eyeglasses. The scene camera was angled downward so that the entire tabletop area was visible. This was followed by a calibration procedure using the Pupil Calibration Marker v0.4 (8 cm diameter), which was placed at four locations on the table: in front of the participant, in front of the other participant, and on the left and right sides of the table, ensuring that the full surface was sampled. Participants were instructed to make several circular head movements while fixating on the marker on the table. The same procedure was then repeated for the other participant.

Afterward, the actual recording of the session began. We recorded touching and untouching events using the touch sensors (switches in the gloves) and hand movement patterns using a multi-camera system (see below), as well as the eye movements of both participants simultaneously. During each session, participants engaged in playing the ACA game. Each session consisted of four blocks, each with 20 action–counteraction pairs. In every block, both players assumed both roles (Leader for 10 pairs, Follower for 10 pairs), with order counterbalanced across participants. Once all four game blocks were finished, the session ended. Each block lasted approximately 3 to 5 min, resulting in a total session time of less than 20 min. In total, 120 data sets were recorded (30 pairs × 4 blocks). Of these, 110 were valid; 10 were excluded due to recording failures.

### Recording setup

#### Overview

**Figure 3:**
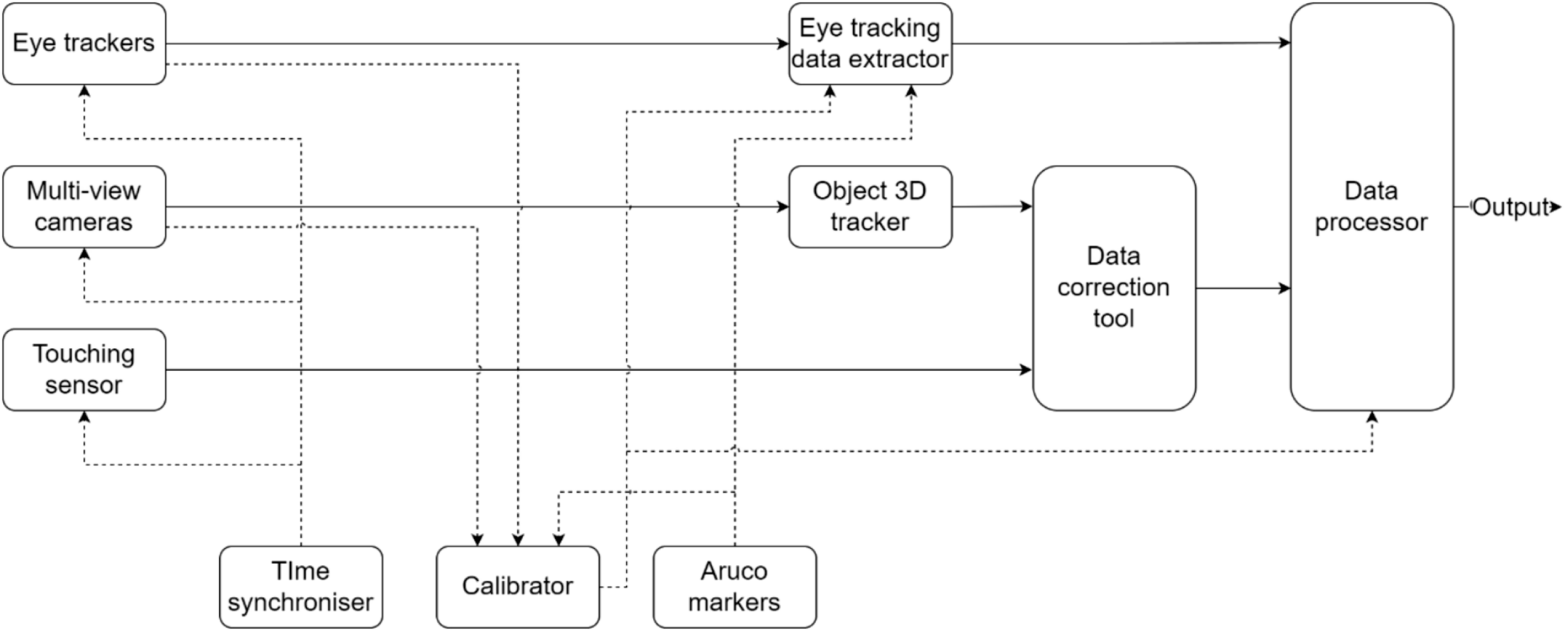
Block diagram of the experiment’s technical setup.

As depicted in Figure 3, the system consists of three input data sources, namely the five-view camera setup based on Teledyne FLIR Grasshopper3 GS3-U3-32S4C-C visible-light cameras, the two Pupil Core eye trackers from Pupil Labs (Kassner et al., 2014), and the touch sensors. They were synchronized using a universal clock, and jointly calibrated using Aruco markers (Apritag 36 h11 family, Garrido-Jurado et al., 2014). The touch sensor was designed to capture the touching/untouching events between hands and objects (T/U events).

In the five-view camera setup, four cameras were mounted at the corners of a 2.5 × 2.5 m square at a height of 1.75 m, each with a downward tilt of 45°. The fifth camera was centered above the table at a height of 2.8 m, pointing straight down. This configuration was tuned so that, in cases of occlusion in one or more views, the remaining cameras would capture the missing details of the interaction. Note that other camera geometries could also be used, provided they adequately minimize occlusions.

The system’s timing and data flow were as follows. Two calibration procedures were performed. First, a general calibration procedure was conducted. During this process, the experimenter generated a 3D representation of the tablecloth filled with Aruco markers using the Pupil Labs eye tracker and associated recording software. Subsequently, a series of images of the same tablecloth was captured by the five-view camera setup, allowing for the generation of a corresponding 3D model. The calibration software was then used to compute the transformation matrix between the five-view camera configuration and the eye trackers. This calibration procedure only had to be done once unless there are alterations in the physical positioning or orientation of the table or cameras. Second, for each experiment, the experimenter calibrated the eye trackers for both participants as described above, and then triggered the start of the recording. The incoming video stream from the five-view camera setup was encoded and written to the hard drive in real-time, alongside the data acquired from the eye trackers and the touch sensors. The recorded data were analyzed offline. During this process, an object tracker identified and tracked objects and hands, and also extracted the head direction present in each video frame. Finally, the data of interest were extracted and analyzed. More information on the recording setup can be found in Cheng (2024).

#### Hand tracking

The hands of the participants were tracked using an Axis-Aligned Bounding Box (AABB) pipeline. Using the recorded camera images from all five cameras, a custom-trained Deep Neural Network (DNN)—YOLOV5 model (Jocher et al., 2020) detected hands in the images and outputs 2D bounding boxes. Subsequently, a triangulation algorithm transformed these 2D bounding boxes into 3D AABBs. Next, the 3D AABBs were tracked using a modified Unscented Kalman Filter (UKF) and finally smoothed with a low-pass Finite Impulse Response (FIR) filter. See Figure 2A for a view of the scene that includes a 3D AABB on one of the objects.

#### Object detection

Object detection and label assignment used the same Yolov5 model. The labels of the different objects were assigned to the various possible object positions (see below) after the completion of a manipulation action.

#### Eye-tracking data extraction

In this study, the eye tracking data of each player were defined as an array of eye fixations over time. Each array entry contained: the starting time of the fixation; the duration of the fixation; the coordinates of the target position, or the instance ID of the target or hand the participant looked at.

Each eye tracker featured three mounted cameras: one scene camera and two eye cameras. Using the recording software provided by Pupil Labs, the eye tracker output the gaze in the form of 2D coordinates on the scene camera and videos from all the cameras. Two types of data were extracted offline after recordings using the software provided by Pupil Labs. One type was the fixation data, which is defined as a set of consecutive frames where the gaze remains at the same point for a sufficiently long period. To determine a fixation, a threshold of +/−2.45 degrees of visual angle was set. Furthermore, a resolution of 20 ms for fixation shifts was chosen such that the initial sampling rate of the eye-movement data was 50 Hz. A fixation onto an object was defined when at least 10 such samples in a row hit the same object; hence, we assumed a minimal fixation duration of 200 ms (Johansson et al., 2001; Pannasch et al., 2008). One should note that small saccades that moved gaze outside the ±2.45° window or broke the 200-ms continuity criterion were not counted as fixations on that object.

The second type of data extracted by the Pupil Player software was the head pose data. By detecting the Aruco markers on the table and employing the Perspective-n-Point (PnP) algorithm, we determined the extrinsic parameters of the eye-tracker’s scene camera. A standard “Headpose Tracker” plugin from the “Pupil Capture” was used. With the head pose data, the 2D gaze fixation was transformed into 3D, which was essential for integrating eye tracking data with the location of the objects.

#### Determining hand and object location

To calculate which hand or object location the eye fixations struck, a ray-tracing algorithm based on the principles outlined in Williams et al. (2005) was implemented in this study (see Supplementary material). Ray tracing essentially implemented a collision-detection algorithm between a virtual ray originating from the eye and each object, determining which object was being fixated.

To conveniently describe the positions of the objects as well as empty target positions, a discretized coordinate system of the table and a set of 18 corresponding virtual 3D AABBs was defined. This was possible because no other object locations were permitted in this game. In Figure 2A, the defined positions were based on the two rows of Aruco markers on the table. The top left corner of the marker corresponds to the defined location with coordinates (0, 0), and the bottom right corner corresponds to (8, 1). Notably, z-coordinates were not required for the present analyses. There were two reasons that virtual 3D AABBs were used in this study. First, they were used to detect whether the participants looked at empty positions on the table. Second, since the objects were relatively small in size, using the virtual 3D AABBs allowed more precise determination of which object the participants looked at, as the objects could only be placed on these defined, discretized positions. Note that object labels could be assigned to the different locations following object detection as described above.

Figure 2B illustrates an exemplary eye-movement track from one of the sessions, demonstrating the basic data structure used for all statistical analyses. As mentioned, objects are arranged in rows 1 and 2 and numbered from 0 to 8 (left to right) in row 1 and from 9 to 17 in row 2, with the diagram truncated above 13 because no saccades occurred to objects 14–17. For this participant, row 1 lay directly in front, and row 2 was further back. The track shown here contains a total of 25 saccades to different objects over a period of 17.5 s. Minimal fixation duration was 220 ms (marked by * near 11.0 s in Figure 2B) and maximal duration 1,500 ms (** at 7.5 s and after 12.5 s). Clearly visible are two progressive sequences of saccades along row 1 (dashed arrows), interrupted by looking at row 2 for a short time. During these progressions, alternating saccades to neighboring (or close-by) objects, highlighted by the ellipses, are found.

### Data analysis

Data were analyzed in two ways: (1) with a time-resolved approach and (2) based on statistical modeling.

### Methods for time-resolved analysis

The raw eye- and hand-tracking data were analyzed with several methods. For hands we determined two data points: (1) the start of the hand movement, determined from the camera images as the moment when the hand leaves the “home” position for more than 5 cm and (2) the moments when the hand touches (T-event) or releases an object (U-event) as well as the durations in-between, determined from the sensor data. T-events and U-events allow the structuring of the eye-fixation analysis of the free-running game, as explained in a Supplementary Video “Presentation_histograms.mp4”.

#### Chunking

**Figure 4:**
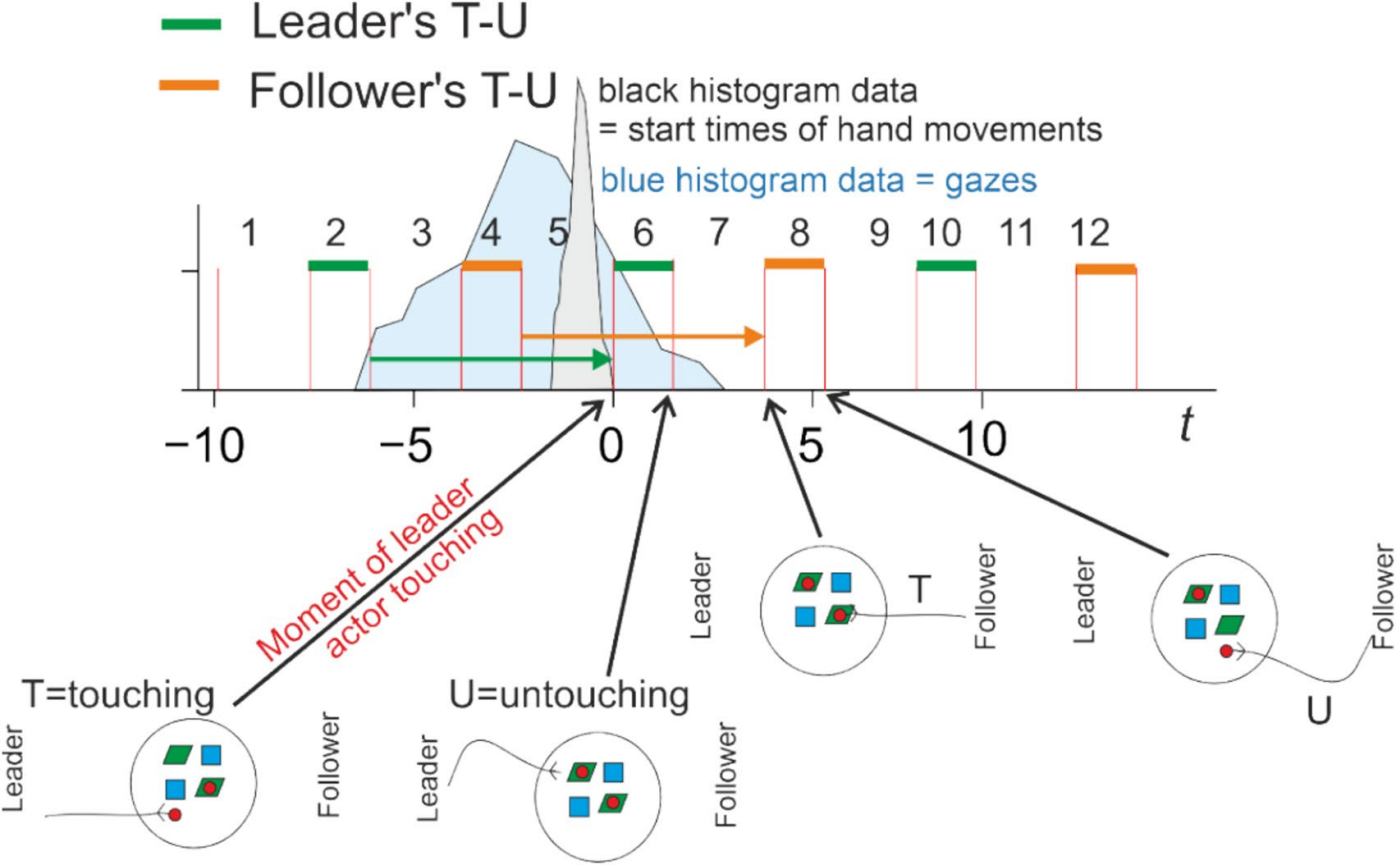
Schematic illustration of the way temporally resolved data are presented. The time axis is labeled in seconds. The time intervals during which the leader or follower is manipulating different objects are shown by green (leader) and orange (follower) bars. The vertical lines indicate touch events (at the beginning of the green and orange intervals) and release events (at the end of those intervals). Between the bars, there are intervals during which both the leader’s and the Follower’s hands are empty (no object in hand). A green or orange arrow indicates the interval during which the Leader’s or Follower’s hand is free. The intervals are numbered consecutively from 1 to 12 above the time axis. We schematically depict two histograms: (1) in blue, fixations on a specific object that is touched at time zero (see the zero tick on the time axis). Note that in the same time intervals, there would also be fixations on other objects, but these are not shown in the Figure. (2) In grey, the onset of the hand movement that will result in touching the object. The time-resolved analyses presented in Figures 5, 6 use intervals 1 to 10 for the leader and 3 to 12 for the follower. Bayesian modeling of predictive fixations uses intervals 3 to 5 for the Leader and 5 to 7 for the follower, for modeling with respect to objects, and intervals 3 to 6 and 5 to 8 (for leader and follower, respectively) with respect to destination. Pictograms illustrate the steps of the experiment. For a step-by-step explanation, see the Supplementary Video “action_counteraction.mp4”.

Observing the aforementioned T- and U-events resulted in a natural chunking of the eye-fixation data streams of the two players. In Figure 4, which schematically represents the time-resolved data, these intervals are indicated by green and orange bars. The green bar starts at the moment when the Leader touches the object and ends when the Leader releases the object at a destination. For the Follower, the corresponding intervals are shown by orange bars. Note that, by design, T-U intervals alternate between Leader and Follower, and there are intervals in between, called object-static intervals, which represent periods when no object is moved (although the hands may still move).

Note that the period where the Leader does not move an object stretches from the end of interval 2 to the beginning of 6 (for follower: end of 4 to beginning of 8), always bridging three intervals. The green and orange arrows indicate this “bridge.” This repeats for other groups of intervals in the same manner (e.g., 1 → 3, 7 → 9, etc.).

#### Episode definition and analysis

**Figure 5:**
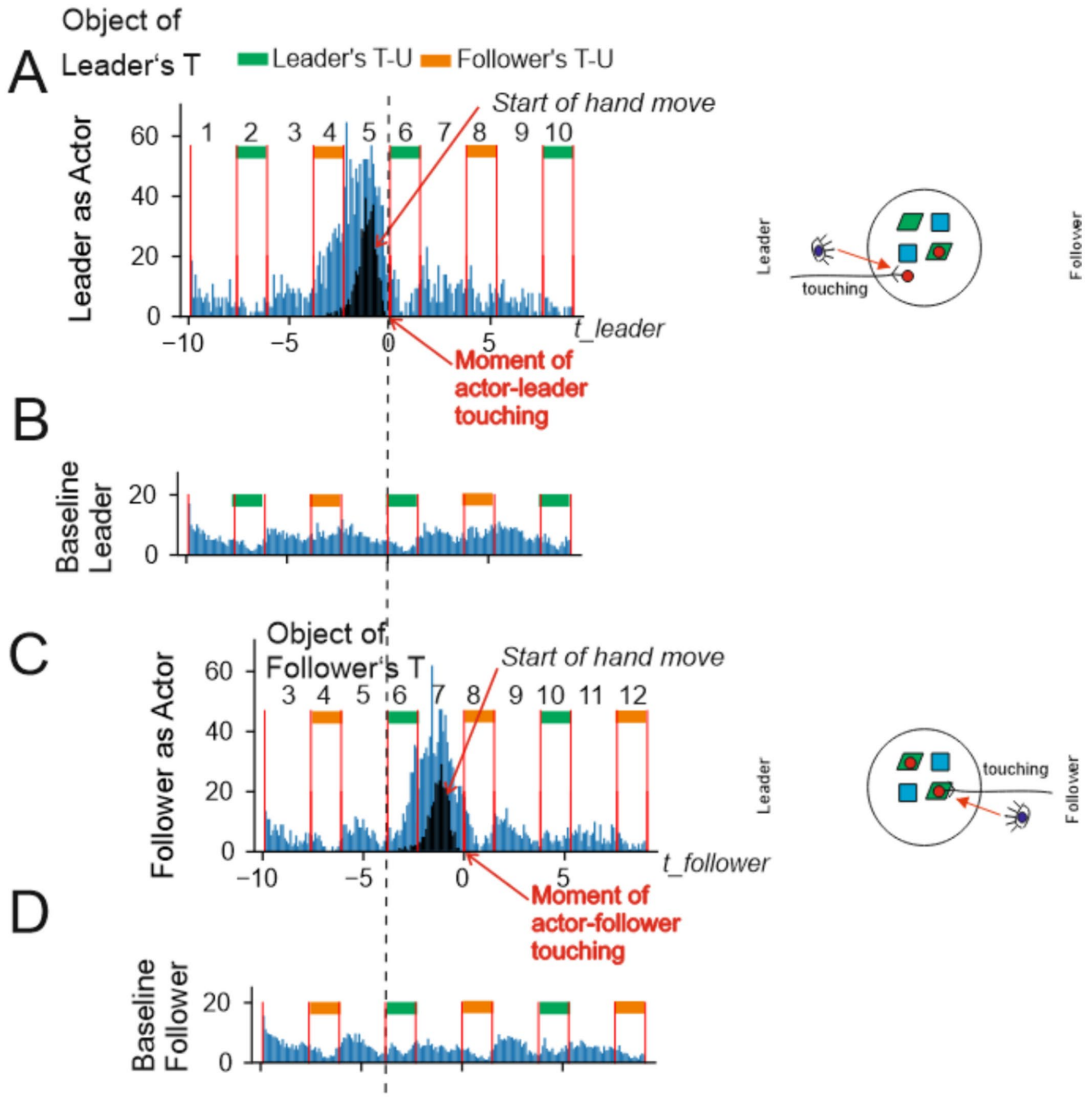
Histograms for two situations and corresponding baselines. Here and in the following: when a diagram is labeled “object” (panels **A,C**) it represents the gazes at that particular object that is being manipulated during this episode and similarly for “destination” (see Figure 6) of where that object was put down. Histograms are calculated to represent the number of fixations onto object or destination over all participants and all objects/destinations, where the ordinate is normalized by the number of episodes analyzed (see method section). On the right side, a schematic shows the table configuration looks like at the given moment in time and also what happens for leader and follower (touching an object, untouching the object after placing it at a new destination). Hence, panel **(A)** left side shows the situation where the leader touches an object (pictogram of the small red disk) and the histogram(s) show the start of the Leader’s hand movement (black) and – as indicated by the eye pictogram – the statistics of the Leader’s gazes at this object (blue). In **(B)**, one can see the baseline gaze distribution (i.e., gazes to non-manipulated objects in the interval) for the same period shown in **(A)**. Panel **(C)** depicts the next interval used to analyze the follower’s gazes. Finally, in **(D)**, one can see the distribution of baseline gazes (i.e., gazes to non-manipulated objects in the interval) for the same interval as depicted in **(C)**. Diagrams are labeled by different action- as well as inactivity-intervals (numbers and orange, green markers).

**Figure 6:**
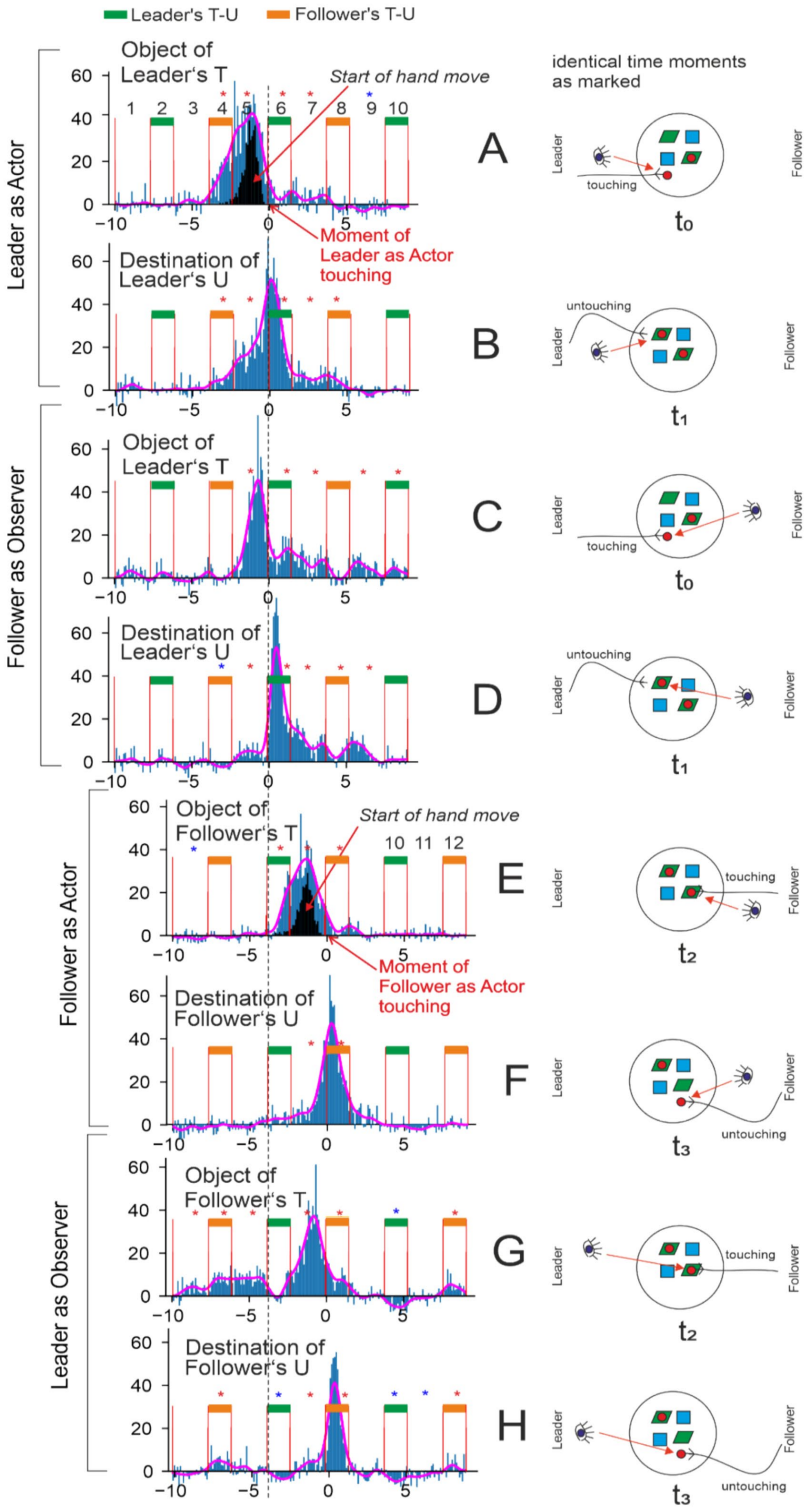
Detailed temporal analysis of hand and eye-fixation patterns. Eye-fixation histograms (blue) are shown after subtraction of the baselines indicated in Figure 5. A histogram indicating the onset of the actor’s hand movement is shown in black. Green and orange bars mark intervals in which the Leader’s or follower’s hand holds an object. In intervals without bars, both hands are free. The magenta lines show a smoothed envelope of the blue histograms. Red and blue asterisks indicate *p* < 0.05 in a one-sided Wilcoxon test for the conditions “count > 0” and “count < 0,” respectively. Pictograms depict the condition for each histogram: who (Leader or follower) is acting, and whose (Leader’s or follower’s) fixations are shown. Touch versus release is also distinguished in the pictograms. **(A–D)** Fixation histograms for actor–leader and follower–observer. **(E–H)** Fixation histograms for actor–follower and leader–observer. In **(A)**, fixations of the actor–leader on the object touched by them at the beginning of interval 6 are shown. In **(C)**, fixations on the same object by the observer are shown. In **(B)**, fixations of the actor–Leader on the destination used at the end of interval 6 are shown. In **(D)**, fixations on the same destination by the observer are shown. In **(E)**, fixations of the actor–follower on the object touched by them at the beginning of interval 8 are shown. In **(G)**, fixations of the observer on the same object are shown. In **(F)**, fixations of the actor–follower on the destination used at the end of interval 8 are shown. In **(H)**, fixations of the observer on the same destination are shown. The dashed vertical line marks the moment when the actor–Leader touches the object in episode **(A)**. This line facilitates understanding of the relations between the Leader’s actions in episodes **(A–D)** and the follower’s actions in episodes **(E–H)**. For example, in **(G)**, the Leader is the observer of the object manipulated by the follower, and the dashed line helps indicate when the Leader’s fixations on that follower’s object occur relative to the Leader’s own action.

A total of 12 intervals, schematically represented in Figure 4, is shown when presenting the results of the time-resolved analysis (Figures 5, 6). For this purpose, we define an episode as consisting of 10 intervals in which the fixations of a player are analyzed. Episodes comprising intervals 1 to 10 (out of 12) were used to analyze the Leader’s actions, and episodes comprising intervals 3 to 12 were used to analyze the Follower’s actions. We analyzed fixations with respect to one specific object in each analysis run. For the Leader’s actions, we first analyzed their fixations with respect to the object manipulated at the start of interval 6 (as numbered in Figure 4, top). We then conducted the same type of analysis with respect to the destination, i.e., the location at which the object manipulated in interval 6 was placed by the Leader. The same two analyses were performed for the Follower as observer, again with respect to the same object manipulated by the Leader. The Follower’s counter-actions occur later (after interval 6). Accordingly, for the Follower as actor, we analyzed their fixations on the object and the destination related to interval 8. As a result of this procedure, a total of eight analyses were carried out for all 2 × 2 × 2 conditions of (leader/follower), (actor/observer), and (object/destination). For each analysis (one episode, one object), only a small number of fixations is available. Therefore, the above-described procedure was repeated across many episodes, and histograms were formed (see below).

#### Episode validity

In this procedure, care was taken to ensure that the analyzed object or destination was not used twice in the same episode. Episodes in which this occurred were excluded from the analysis, because once the same object, say O1, was manipulated twice, it was no longer clear to which of the two manipulations fixations on O1 should be attributed. As a consequence, the number of valid episodes differed across conditions. The number of valid episodes for all eight combinations of (Leader/Follower), (actor/observer), and (object/destination) ranged from 650 to 1,143, with an average of 850.

#### Histogram formation

Based on the valid episodes, eight histograms of fixation counts were formed for all combinations of (Leader/Follower), (actor/observer), and (object/destination). To account for differences in the number of valid episodes and to make the data comparable across the different analyses, we normalized all histograms shown in the time-resolved analysis (Figures 5, 6) to 1,000 episodes.

#### Baseline

For the baseline, we constructed histograms of fixations on objects that were not manipulated during an episode, while preserving the object manipulation frequencies exhibited by each pair of participants in a given experimental session. To achieve this, we first obtained the distribution over all 18 possible object locations by counting how many times an object at each location had been manipulated during the current experimental session. For the episode under consideration, we then excluded from this distribution all objects that had actually been manipulated. The resulting distribution was normalized to 1, yielding a probability density. Next, we drew an object location from this density in the conventional way. This draw yielded the (non-manipulated) object to be considered for gaze analysis in that episode. For each episode, five such draws were made, and the number of gazes onto these objects was counted and accumulated in the corresponding baseline histogram bins. A total of 800 episodes were treated in this way. Because the “same non-manipulated object” occurred randomly in different intervals of different episodes, this procedure effectively permuted the set of objects not manipulated in the episode, using information from the entire session. This was done separately for the Leader and the Follower.

#### Hand movement

The black trace in Figure 4 schematically encodes the histogram of the onset times of the actor’s hand movements. The actor’s hand movements are important for analyzing the observer’s fixations on manipulated objects, as the observer can predict in advance (i.e., before actual contact) which object will be grasped by the actor based on the hand trajectory.

#### Temporal normalization

Given that the duration of the intervals was not constant but varied from episode to episode and between different players, we normalized all interval durations in the histograms (Figures 5, 6) to their respective averages, which were 1.5 s for T–U intervals and 2.3 s for object-static intervals (Leader and Follower alike), and we performed time-warping of all the temporal gaze data within each interval. In a free-running (no triggers, no time limits) experiment, such as the one presented, players were sometimes inattentive or distracted, or for other reasons, overly long or short interval durations would occur. Hence, extreme outliers were removed [ Quartile1(Q1)−3∗interquartile(IQR),Q3+3∗IQR ]. We found that about 5% of intervals fell into this category.

### Statistical methods

The data were processed and analyzed using the R programming language.^1^ First, search zones were defined for extracting the relevant gazes for each event (arrows in Figure 4). For Touching and Untouching events, the search zone started from the event and extended until the previous Untouching of the same kind (e.g., the Touching of an observer to the previous Untouching event of the observer). Additionally, filter zones were defined that included the two previous Untouching events of the same kind. To ensure interpretability, all events with two similar target gazes in the filter zone were excluded from the final analyses. This procedure resulted in the elimination of approximately 25% of the events.

The selection of search and filter zones was based on the fact that participants could start planning an action (i.e., grabbing an object and moving it to the chosen destination) only after completion of the previous action, i.e., the previous Untouching. However, as soon as they started planning the action, they could simultaneously plan which object they wanted to move and where they wanted to place it. Therefore, the zones started from the previous Untouching event for both the Touching and Untouching conditions. Furthermore, as planning could only take place before an event, for Touching, the search and filter zones could only extend from the previous Untouching event to the Touching event; however, for Untouching, they would extend from the previous Untouching event to the next Untouching event. To control for the effects of longer search zones for Untouching compared to Touching events, we added the duration of search zones to our models as a nuisance regressor.

For each event, three variables were calculated. (1) The number of correct fixations, defined as the number of matches between the current fixation and the target destination for the event in the search zone. Specifically, the correct fixation for a Touching event was a gaze directed to the location where the object to be moved was located. The correct fixation for an Untouching event was a gaze at the final destination of the object to be moved. (2) The length of correct fixation, defined as the cumulative duration of correct fixations for each event. Finally, (3) the fixation latency, defined as the time interval between a correct fixation and the end of the event. Notably, for each event, several fixation latencies could be extracted, in contrast to the count and length variables, which only had one value per event. Therefore, the latency of each correct fixation for each event was entered into the model separately.

Each event had three categorical properties that were modulated within-subjects: (1) Activity: Actor vs. Observer, (2) Role: Leader vs. Follower, and (3) Event: Touching vs. Untouching. The response variable for each cell of the design was checked for any extreme outliers in the same way as above [i.e., outside Quartile1(Q1)−3∗interquartile(IQR),Q3+3∗IQR ]. Since the response variables were not modeled under the assumption of a normal distribution, outliers, when present, were not excluded; instead, other techniques, such as using robust statistics and weighted models, were employed to mitigate their effects (Agresti, 2015; Huber and Ronchetti, 2011) and are described in the text. Bayesian generalized hierarchical linear regression models (BGLM) were used to investigate each of the variables. For each response variable, the three explanatory variables and their interactions were included in the models. Based on recent developments in statistics (Rouder et al., 2022; van den Bergh et al., 2023), we employed the maximum random-effects model. Thus, we assumed random intercepts and slopes for all included main and interaction effects for each participant (i.e., $\mathrm{su}b_{\mathrm{id}}$ in Equation 1). Finally, to control for differences in search zone length across various events, a search zone length variable was added to the model as a nuisance regressor.


$$\begin{array}{l} \text{Outcome}\sim \text{Actor}*\text{Leader}*\text{Touching}+\text{Lengt}h_{\text{Event}}+\\ \left(\text{Actor}*\text{Leader}*\text{Touching}\;|\mathrm{su}b_{\mathrm{id}}\right) \end{array}$$ (1)

For calculating Bayesian hierarchical generalized linear models, the packages brms (Bürkner, 2017) and RStan^2^ were employed. All models were estimated using five chains, each with 4,000 iterations and 2000 warm-up iterations. If any variable showed $\hat{R}$ , the potential scale reduction factor on split chains, above 1.05, the model was recalculated with increased iterations, and the results were reported accordingly. Finally, since all the models were hierarchical, weakly informative priors were preferred (Rouder et al., 2022; van den Bergh et al., 2023). The exact weakly informative priors used for each model are described below.

For modeling the fixation counts (Equation 2), the response variable represented the number of events occurring within a specific time window. Hence, a negative binomial BGLM was employed. A negative binomial distribution was preferred over a Poisson distribution due to overdispersion. The weakly informative priors for the model were as follows: N(0,10) for the intercept, N(0,2) for the *β* coefficients, Student_t(3,,0,,2.5) for the *σ* and SD hyperparameters, inv−Gamma(0.01,0.01) for the shape hyperparameter, and lkj(1) for the correlations between random variables.


$$\begin{array}{c} \begin{array}{l} \text{Fixatio}n_{co\mathrm{unt}}\sim \text{Actor}*\text{Leader}*\text{Touching}+\text{Lengt}h_{\text{Event}}+\\ (\text{Actor}*\text{Leader}*\text{Touching}\;|\mathrm{su}b_{\mathrm{id}}), \end{array}\\ \text{family}=\text{negbinomial}(\text{link}=\;“\log”,\;\mathrm{lin}k_{\text{shape}}=\log) \end{array}$$ (2)

For modeling fixation length (Equation 3), a hurdle-lognormal model was used (Cragg, 1971; Neelon et al., 2010). The hurdle model allows us to handle zeros (i.e., when no correct gaze was found and thus the gaze length was zero) simultaneously with the rest of the data (lognormal data related to gaze length when there was at least one correct gaze). The weakly informative priors in the models were: N(0,10) for the intercept, N(0,2) for the β coefficients, Student_t(3,,0,,2.5) for the σ and SD hyperparameters, and lkj(1) for the correlations between random variables.


$$\begin{array}{c} \begin{array}{l} \text{Fixatio}n_{\text{length}}\sim \text{Actor}*\text{Leader}*\text{Touching}+\text{Lengt}h_{\text{Event}}+\\ (\text{Actor}*\text{Leader}*\text{Touching}\;|\mathrm{su}b_{\mathrm{id}}), \end{array}\\ \text{family}=\text{hurdle}\_\text{lognormal}(\begin{array}{l} \text{link}=“\text{identit}y”,,\text{link}\_\text{sigma}=\\ “\log”,,\;\text{link}\_\mathrm{hu}=“\text{logi}t” \end{array}) \end{array}$$ (3)

Finally, we used a lognormal model to investigate fixation latency. The weakly informative priors employed for this model were: N(0,10) for the intercept, N(0,2) for the β coefficients, Student_t(3,,0,,2.5) for the σ and SD hyperparameters, and lkj(1) for the correlations between random variables.


$$\begin{array}{c} \begin{array}{l} \text{Fixatio}n_{\text{latency}}\sim \text{Actor}*\text{Leader}*\text{Touching}+\text{Lengt}h_{\text{Event}}+\\ (\text{Actor}*\text{Leader}*\text{Touching}\;|\mathrm{su}b_{\mathrm{id}}), \end{array}\\ \text{family}=(\text{link}=“\text{identit}y”,\text{link}\_\text{sigma}=“\log”) \end{array}$$ (4)

For the model comparison, we used the Pareto smoothed importance sampling (PSIS) estimation of leave-one-out cross-validation (LOO) implemented in the LOO package (Magnusson et al., 2020; Vehtari et al., 2016). LOO assesses pointwise out-of-sample prediction accuracy from a fitted Bayesian model using the log-likelihood evaluated at the posterior simulations of the parameter values; however, as it was difficult to calculate, importance weights ware commonly used instead, resulting in PSIS-LOO. To make sure that PSIS-LOO estimation was accurate, we used $\text{Pareto}\;\hat{k}<0.7$ . However, $\text{Pareto}\;\hat{k}<1$ was still considered acceptable.

All hypotheses were tested using the function in brms (Bürkner, 2017). Based on the suggestion of van Doorn et al. (2021), Bayes factors(BF)>3 were considered as credible evidence for the tested hypothesis. One-sided hypotheses ( $BF_{+0}$ and $BF_{0+}$ ) compared the posterior probability of a hypothesis against its alternative. On the other hand, two-sided tests ( $BF_{10}$ and $BF_{01}$ ) compared hypotheses with their alternatives using the Savage-Dickey density ratio method (Bürkner, 2017).

#### Validity

In all cases, the different model variants converged correctly without any divergent transitions (all Rhat=1 ). Furthermore, the bulk and tail effective sample sizes for main effects and interactions were each above 4,000, indicating that model predictions were reliable. The model accurately captured the observed data distribution.

## Results

All analyses used a minimal fixation duration of 200 ms, which is realistic threshold in this context (Johansson et al., 2001; Pannasch et al., 2008). Note that we annotate with “object” the grid location of an object about to be manipulated by the actor. We use the same annotation “object” for grid locations from which an object has been removed. With “destination,” we annotate the place where an object was or would be placed. The latter includes all possible places on the game grid (not only empty ones).

First, we provide a time-resolved analysis of general effects, followed by detailed statistical analyses to consolidate the main findings.

### Temporal characteristics of the viewing behavior

Figure 5 shows the viewing behavior as blue histograms alongside hand-movement onsets (panels A,C, black histogram). Histograms were centered (“zero”) at the moment where the leader (A,B) or the follower (C,D) touched the object, which is to be manipulated. This was chosen because most gazes were expected—and indeed observed—before this moment as shown by the main blue peaks. Detailed evaluations are provided (Figure 6). The dashed vertical line visually aligns the top and bottom panels.

When the Leader was the actor (A), they *started* their hand movements during interval 5 (black histogram) and the hand then eventually touches the object. The blue histogram shows the distribution of the Leader gazes at the object being manipulated at this stage. The bulk of this occurred before interval 6. Hence, as expected, eye movements predict hand movements. In addition, there was a tail that extended into intervals 7–9, which will be discussed later. Panel (B) shows a baseline. It contains all gazes of the participants at objects not having been manipulated during the length of the episodes contained in panel (A). Below we used the baseline to calculate statistical significances.

Panel (C) shows the corresponding situation when the Follower was the actor, with the baseline given in (D).

Hence, in summary, both histograms A and C represent the gazing behavior at the to-be-manipulated objects, illustrating part of the do–undo sequence: specifically, the object-centered viewing behavior of the Leader who prepares and performs an action and the (also object-centered) viewing behavior of the Follower who then acted to perform the undo action. In Figure 6, we now discuss these and more cases in more detail, showing which intervals display a significant deviation from baseline and which did not. We thus subtracted the baselines from their corresponding original plots, which could lead to negative numbers, too. Red (blue) asterisks represent intervals that were significantly greater (smaller) than baseline level (*p* < 0.01 by a one-sided Wilcoxson signed rank test).

Pink curves represent a low-pass filtered version of the blue histograms, using a Gaussian filter with a standard deviation (STD) of 0.3 s. Note that diagrams come in pairs occurring at the same time (t_0_, t_1_, t_2_, t_3_, indicated under the pictograms). The vertical dashed line marks the start of interval 6, which is the one where the Leader actually starts their action. All four top diagrams (A–D) show a tail behind the main peak. These diagrams are all about the Leader’s object (taking and placing). These tails represent looks “into the past,” either at the place where the object was located before manipulation and/or at the destination previously covered by the object.

We now provide results in more detail for the different figure panels in Figure 6.

Panel (A): This panel illustrates the behavior of the Leader when considering the to-be-manipulated object. It also includes the start of the Leader’s hand movement, which occurred in interval 5 before the touching of the object. The Leader’s gaze shifted to the object just before interval 4, and mainly during intervals 4 and 5. Meanwhile, in interval 4, the Follower continued their task, undoing the previous game event – an action that does not require the Leader’s attention. Intervals 6 and 7 displayed a tail. Intriguingly, at/after their untouch, some Leaders looked again at the location where the object had been taken away from.

Panel (B): This panel illustrates the behavior of the Leader when considering the destination for placing the object. The Leader looked at the destination very often before touching their object (earliest start of looks at end of interval 3). The whole plot was shifted by one interval relative to panel A, which is expected, because targeting a destination must come after targeting an object. Again, a tail was present, lasting until interval 8.

Panel (C): This panel illustrates the behavior of the Follower who observed the object that is later selected by the Leader. Naturally, the Follower looked at the Leader’s object *only* when or after the Leader actually had started to move the hand [compare peak in (C) to the black histogram in (A)]. There was only a short delay of approximately 400 ms between the peak in panel C and the black peak in panel A. Again, there was a tail of looks at the location where the Leader’s object had been removed from.

Panel (D): This panel illustrates the behavior of the Follower who observed the destination where the Leader placed the object. This observation closely followed interval 6 — the Leader’s action interval — and showed little predictive component. The bulk of the Follower’s gazes occurred just slightly before the Leader’s untouch, and some gazes occurred afterwards. Again, there was a clear longer-lasting after-look tail.

Intriguingly, for diagrams E-H (Follower as actor and Leader as observer) there was no tail after the main peak. Note that at time t3 the initial situation on the table was recovered [compare panels (A,F)].

Panel (E): This panel illustrates the behavior of the Follower when considering the object to be used for the undo action. This diagram resembles a shifted copy of panel A with very much the same characteristics but without the tail. This general shape was expected as here the Follower was preparing to touch their object. Remarkably, when overlaying the smoothed curve of E onto panel A, one finds an almost perfect fit to the main peak in A.

Panel (F): This panel illustrates the behavior of the Follower when considering the destination for placing the object. The same observation as for panel E also held for panel F: It was essentially a shifted copy of panel B without tail. Here, too, the smoothed curve matched the one from above exceedingly well.

Panel (G): This panel illustrates the behavior of the Leader who observed the object that the Follower will take. It shows a somewhat broader peak when looking at the Follower’s object, compared to the Follower’s peak in panel C when looking at the Leader’s object. The main peak occurred before the object is touched by the Follower. By aligning it with the hand movement plot in panel E, it is evident that the Leader uses the hand movement of the Follower to predict which object the Follower will touch. This is again similar to the observation in panel C. However, the main peak in (G) is slightly broader before its maximum compared to the peak in panel C, because the Follower in panel C had no advance information about the Leader’s next action and could only predict based on the Leader’s hand movement. In contrast, the Leader knew that the Follower had only a limited set of possible objects to choose from, which sometimes allowed for an earlier prediction. Furthermore, in intervals 4 and 5 there was a small but significant peak, indicating that – while the Leader was planning their own action (and at the same time observing what the Follower was doing) – they also looked at potential objects the Follower could use in the future to undo their currently planned actions.

Panel (H): This panel illustrates the behavior of the Leader who observed the destination where the Follower placed the object. The histogram was narrow and, quite similar to (D), it began with a slight predictive component during the Follower’s movement (interval 6, orange) and ended with the Follower’s release of the object. A narrow histogram was expected, because, similar to (D), this pattern mainly reflected reactive (non-predictive) observation, occurring sharply in alignment with the Follower’s movement interval (orange).

After exploring the eye and hand movement data, we next applied Bayesian generalized hierarchical modeling to statistically test the differences between different roles, activities, and event types in fixation count, duration, and latencies.

### Fixation count – number of correct fixations, their distribution, and fixation patterns

#### Fixation count

We used Equation 2 to determine the number of eye fixations on objects and destinations during the task and under different conditions. We studied the main effects and interactions via hypothesis testing (for full results, see Supplementary Table 1).

The results showed strong evidence for the interaction between Role and Event (H_0_: Role*Event = 0, *Estimate ± SE* = 0.21 ± 0.05, CI = [0.10, 0.31], *p.p.* = 0.01, *BF_01_* = 0.01). When being a Leader ( $\mathrm{mea}n_{\text{Untouching}-\text{Touching}}=0.35\pm 0.38$ ) compared to a Follower ( $\mathrm{mea}n_{\text{Untouching}-\text{Touching}}=0.33\pm 0.39$ ), the difference in fixation counts between Touching and Untouching events was bigger.

Additionally, strong evidence for the interaction between Role and Activity (H_0_: Role*Activity = 0, *Estimate ± SE* = 0.19 ± 0.05, *CI* = [0.10, 0.28], *p.p.* = 0.00, *BF_01_* = 0.00) revealed that Leaders ( $\mathrm{mea}n_{\text{Actor}-\text{Observer}}=0.34\pm 0.01$ ), compared to followers ( $\mathrm{mea}n_{\text{Actor}-\text{Observer}}=0.15\pm 0.01$ ), had a higher fixation count when acting compared to observing.

**Figure 7:**
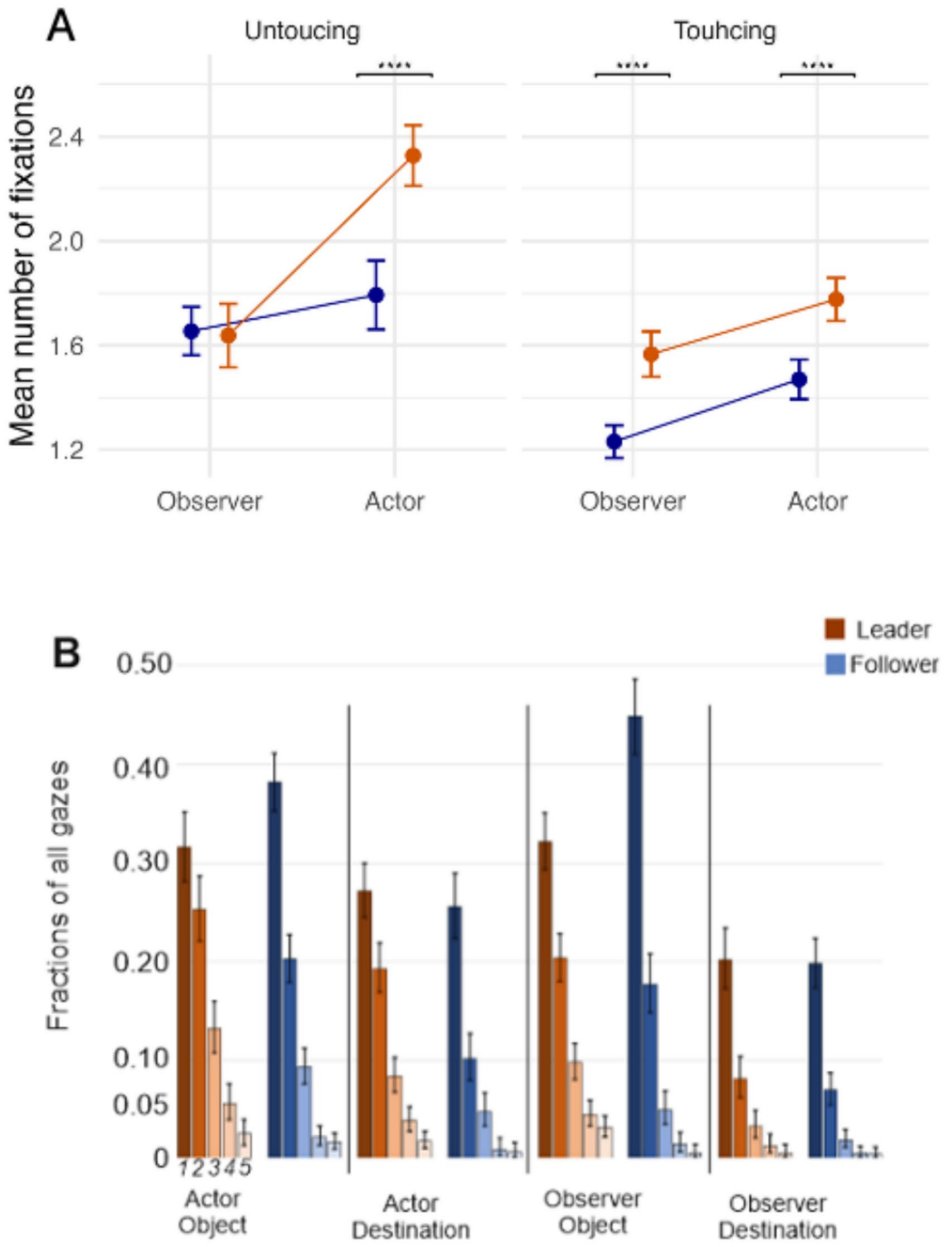
**(A)** Fixation counts for the three-way interaction between leader, actor, and touching factors. Point estimates and error bars represent the mean and confidence interval, respectively (**** *p* < 0.0001). **(B)** Single versus multiple fixations, Bars 1 to 5 represent how often a certain fixation has happened. We use a 95% confidence interval calculated for proportions, where the Clopper-Pearson (exact) method for the Binomial distribution was used.

Finally, also strong evidence for the three-way interaction between role, activity, and event (H_0_: Role*Activity*Event = 0, *Estimate ± SE* = −0.23 ± 0.06, *CI* = [−0.36, −0.11], *p.p.* = 0.05, *BF_01_* = 0.06), indicated that although Leaders compared to Followers looked more frequently at objects moved by themselves or their partners, they looked at the destinations more frequently only when acting themselves, but not when their partner was moving an object (Figure 7A).

Together, these results indicate that Leaders, compared to Followers, looked at the object that they were moving and its destination more frequently, indicating repeated attention and higher saliency (Irwin, 2004). However, the higher interest of Leaders in the destination of objects vanishes when observing the actions of their partner (i.e., the Follower), showing a shift in resource allocation from following a movement with an already-known outcome to planning their own next movement. One should note that the destination of an object moved back by a Follower, after the Leader’s move, was already clear (for more details about the paradigm, check the method section). More detailed information about the underlying distributions is provided in Figure 7B.

### Fixation durations

**Figure 8:**
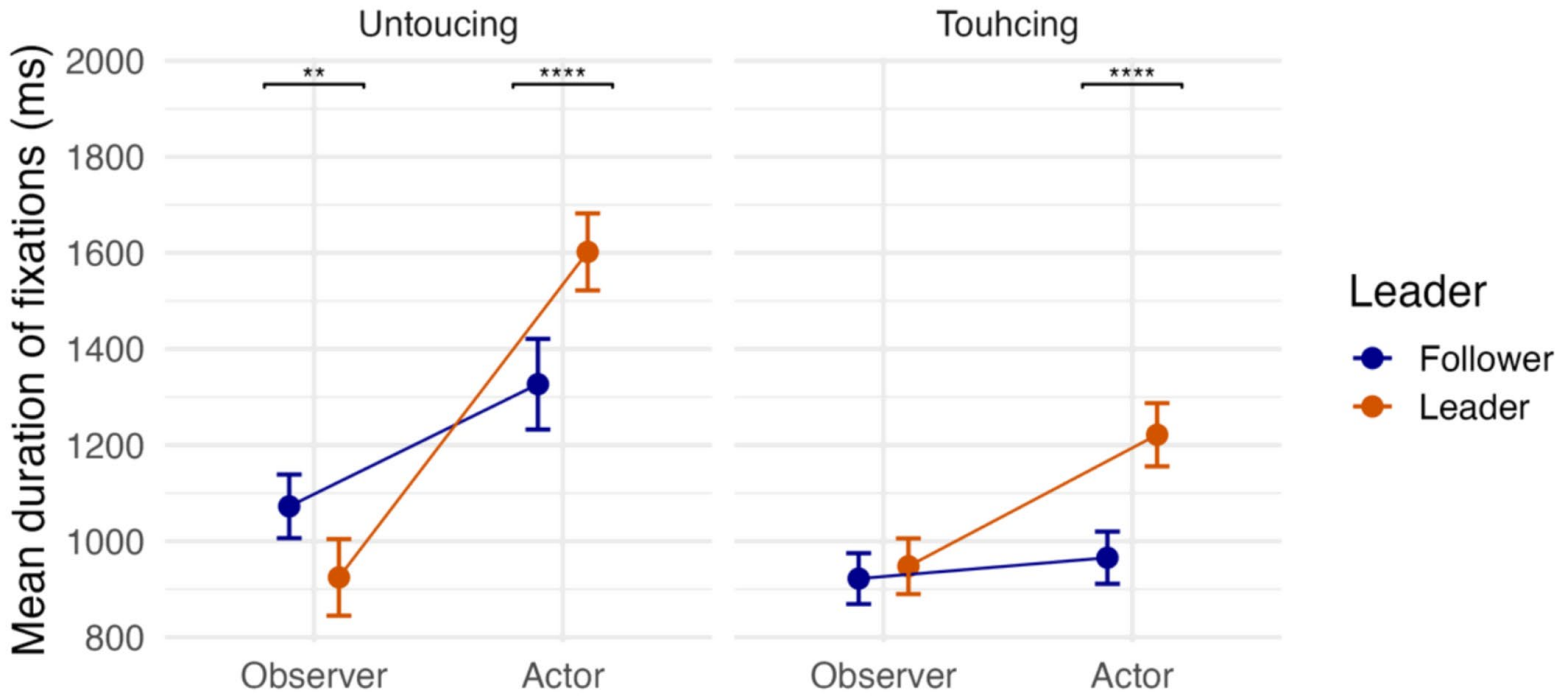
Fixation durations for the three-way interaction between leader, actor, and touching factors. Point estimates and error bars represent the mean and confidence interval, respectively (** *p* < 0.01, **** *p* < 0.0001).

We first focused on fixation length (Equation 3) and investigated the main effects and interactions via hypothesis testing (Figure 8, for the full results, see Supplementary Table 2). The results showed that, when acting ( median=720ms±570 ) compared to observing ( median=653ms±571 ), participants looked at the target (object as well as destination) significantly longer (*H_0_: Activity* = 0, *Estimate ± SE* = 0.18 ± 0.03, *CI* = [0.12, 0.25], *p.p.* = 0.00, *BF_01_* = 0.00).

The strong evidence for interaction between Activity and Event (H_0_: Activity*Event = 0, *Estimate ± SE* = −0.19 ± 0.04, *CI* = [−0.27, −0.11], *p.p.* = 0.00, *BF_01_* = 0.00) revealed that when observing, participants spent more time looking at the actor’s object (i.e., Touching events: median=712ms±628 ) compared to the actor’s destination (i.e., Untouching events: median=570ms±449 ). However, when acting, the trend is reversed, meaning participants spent more time looking at the actor’s destination ( median=912ms±528 ) rather than the actor’s object ( median=626ms±688 ). This interaction reveals that when planning an action to be executed, attention allocation changed qualitatively compared to when observing an action.

Finally, there was also strong evidence for interaction between Role and Activity (H_0_: Activity*Role = 0, *Estimate ± SE* = 0.20 ± 0.05, *CI* = [0.10, 0.29], *p.p.* = 0.01, *BF_01_* = 0.01). This result showed that, when being a Leader, participants were more focused on their own objects and destinations ( median=741ms±685 ) rather than the Followers ( median=566ms±465 ). However, when being a Follower, participants attend to the Leader’s objects and destinations ( median=782ms±644 ) more than their own ( median=708ms±554 ). These results depict a leader-centered interaction between pairs.

### Fixation latencies

We investigated fixation latency (Equation 4) to understand cognitive processes related to planning actions, which consist of two parts: moving an object (i.e., Touching events) and placing it at a destination (i.e., Untouching events). Latency here refers to the time between a fixation on an object (or destination) and the touching (or untouching) event.

**Figure 9:**
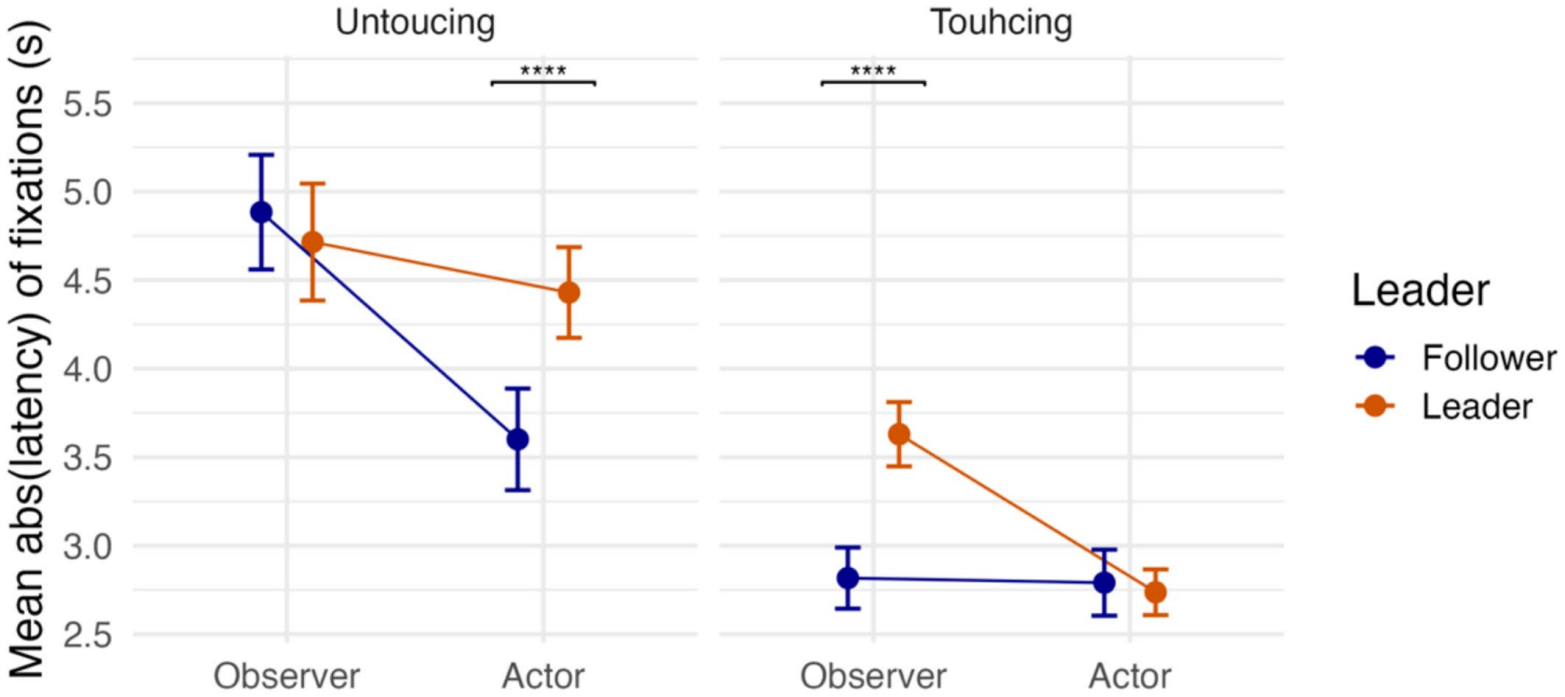
Fixation latencies under different conditions. Point estimates and error bars represent the mean and confidence interval, respectively (**** *p* < 0.0001).

First, we checked the main effects and interactions via hypothesis testing (for the full results, see Supplementary Table 3). The results (*H_0_: Touching* = 0, *Estimate ± SE* = −0.12 ± 0.03, *CI* = [−0.18, −0.06], *p.p.* = 0.04, *BF_01_* = 0.04) showed (Figure 9) that participants looked at the destination ( median=2.59s±5.48 ) significantly earlier than the object itself ( median=1.95s±3.2 ). The strong evidence for the main effect of Role (*H_0_: Role* = 0, *Estimate ± SE* = 0.15 ± 0.03, *CI* = [0.09, 0.22], *p.p.* = 0.00, *BF_01_* = 0.00) further showed that fixation latencies when following ( median=1.85s±4.38 ), compared to when leading ( median=2.48s±4.15 ), were significantly shorter.

Finally, there was moderate evidence for the two-way interaction between Activity and Event (Figure 9) (H_0_: Activity*Event = 0, *Estimate ± SE* = 0.14 ± 0.04, *CI* = [0.06, 0.22], *p.p.* = 0.11, *BF_01_* = 0.12) and Role and Event (H_0_: Role*Event = 0, *Estimate ± SE* = 0.12 ± 0.04, *CI* = [0.04, 0.20], *p.p.* = 0.26, *BF_01_* = 0.32). *Post hoc* tests revealed that when leading compared to following, participants looked at the object to be moved by their partner and the destination of their object significantly earlier. One can understand these results if one notices that the significantly longer fixation latencies for the Leader ( median=2.74s±3.64 ) compared to the Follower ( median=1.50s±3.32 ) in looking at the object that is moved by the partner indicate that the Leader has advanced information about which object is going to be touched by the Follower. However, Leaders ( median=2.77s±4.93 ), compared to Followers ( median=2.04s±5.14 ), look significantly earlier at the destination of the object that they are moving. This result shows that Leaders allocate more resources to planning their actions fully from the beginning compared to Followers (i.e., both the object that should be moved and its destination).

### Sequences

**Figure 10:**
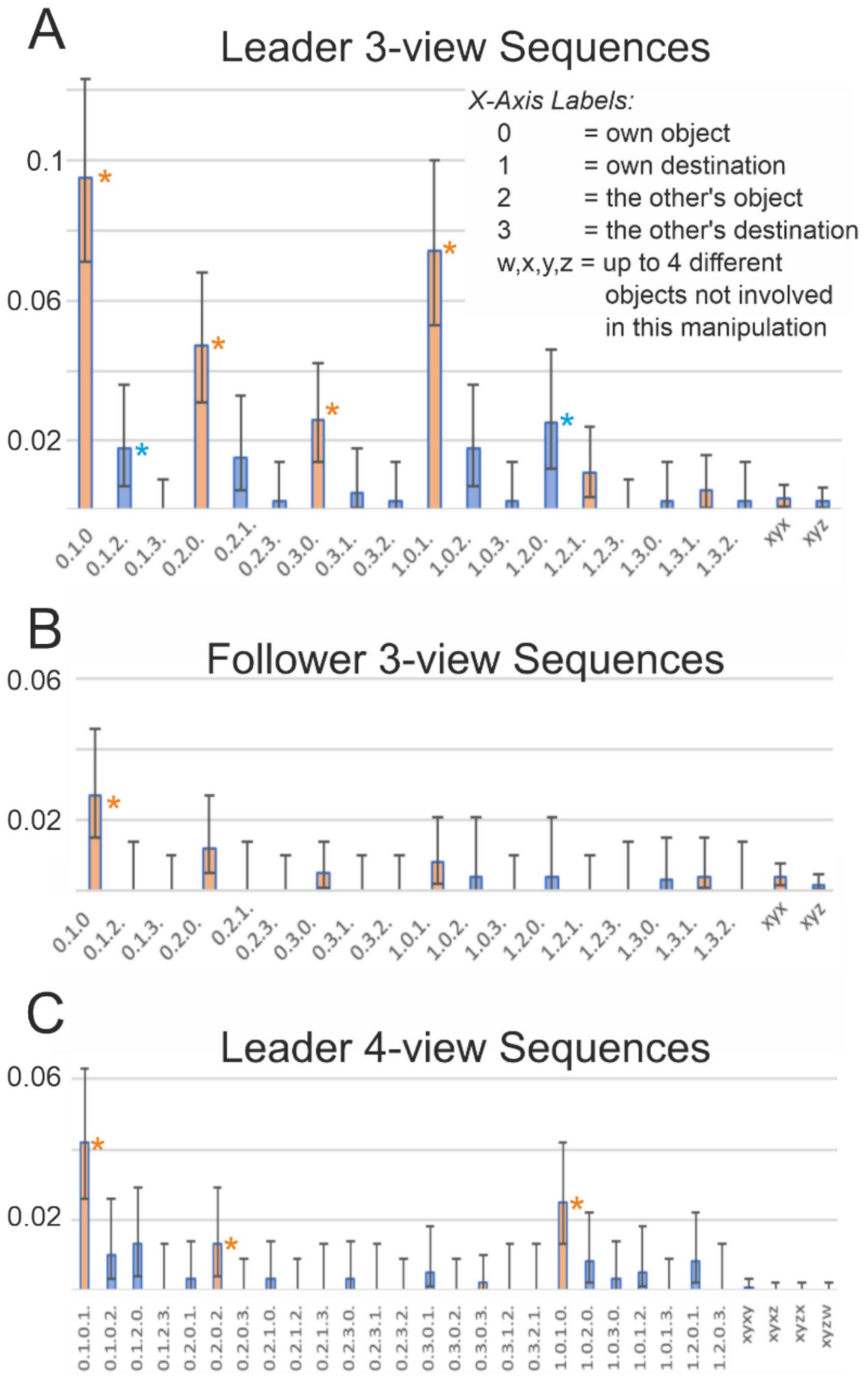
Frequencies of sequence occurrences in the episodes. In each episode, intervals 3 to 6 (as defined in Figure 4) were analyzed for the leader, and intervals 5 to 8 for the follower. Error bars show 95% confidence intervals for binomial distributions. In **(A,B)**, fixation sequences of length three are analyzed: in **(A)** for the leader and in **(B)** for the follower. In **(C)**, fixation sequences of length four are analyzed for the leader. Explanation of the annotation: For example, in **(A)**, the first bar at marker “0.1.0” with a value of 0.095 indicates that actor–leaders fixated the object they were to manipulate (“0”), then the destination (“1”), and then the object again (“0”) in 9.5% of all analyzed episodes, and so on. As baselines, we use sequences of fixations on objects that were not manipulated in the analyzed episodes. Thus, x, y, z, and w denote “some” objects that were not manipulated in the analyzed episodes. For example, the horizontal axis label “xyx” refers to a sequence of fixations in which the same object receives a fixation twice, at the beginning and at the end of the sequence, whereas “xyz” refers to a sequence of fixations on three different objects. Asterisks denote cases in which the frequency of a sequence is higher than that of the corresponding baseline sequence, using a 95% confidence criterion (i.e., the confidence intervals do not overlap).

Finally, we asked whether participants produced sequential combinations of fixations on objects and destinations. Such patterns are particularly informative for understanding decision processes that unfold after a participant has fixated on the object they will soon manipulate—coded on the abscissa in Figure 10 as a leading “0”—or on the destination where that object will be placed—coded as “1.” To examine this, we analyzed potentially relevant 3- and 4-fixation sequences beginning with 0 or 1. For example, a sequence coded as “0.1.0” denotes a look at one’s own object (“0”), followed by a look at its destination (“1”), and then a return to the object (“0”); the corresponding bar in the figure indicates the proportion of such sequences. Indices “2” and “3” refer to the partner’s subsequent targets (object and destination). Figure 10 plots the frequencies of several such sequences, while the final columns provide baseline values derived from gaze sequences directed at objects not involved in the manipulation during the given episode (bars labeled “xyx” and “xyz” in panels A and B). Error bars represent binomial confidence intervals, computed using the Clopper-Pearson (exact) method. The horizontal lines, color-coded by sequence structure, indicate the confidence intervals of the baseline across the diagram. A sequence can therefore be considered above chance when its bar, including the lower confidence bound, lies entirely above the corresponding baseline line.

Leaders (panels A, C) indeed performed several sequences significantly above chance. All combinations of 010 and 101, as well as their 4-fixation counterparts 1,010 and 0101, were overrepresented (note that the 3-fixation sequences are naturally contained within their 4-fixation extensions). Sequences 020 and 0202 were also prevalent, indicating alternations between the Leader’s own object and the partner’s-follower’s object. In addition, a small overrepresentation was found for 030 (Leader’s object–partner’s-Follower’s destination–Leader’s object), although its 4-fixation counterpart 0303 was rare.

Mixed sequences involving three different entities (x, y, z) were observed only for the combination of 0 = own object, 1 = own destination, and 2 = partner’s object. Specifically, the sequences 012, 021, 102, and 120 occurred, with two (012 and 120) just above chance and the other two just below. This pattern suggests at least a tendency for Leaders to integrate their own object and destination with the partner’s object in triadic sequences.

For the Follower (B), the first four orange bars resembled a compressed version of the corresponding bar for the Leader (A). However, only the 3-view sequence 010 was significantly above chance. No 4-view sequences reached significance for the Follower, so this plot is omitted.

Taken together, these findings show that Leaders often linked their own objects and destinations in extended alternations, sometimes incorporating the partner’s objects or destinations as well. Followers, by contrast, showed much simpler sequential patterns, largely limited to alternations between their own object and its destination.

## Discussion

This study investigated how gaze and action unfold in a continuous, role-based interaction. Unlike many laboratory tasks with artificial cues or parallel cooperation, our “do–undo” game required alternating Leader and Follower roles. In this setting, the Follower’s planning necessarily depended on the Leader’s behavior, while the Leader retained more autonomy but could still anticipate the Follower’s next moves. In our setup, the follower has fewer affordances and, thus, a smaller choice entropy. However, note that in everyday joint actions with a follower-leader configuration, smaller choice entropy is an inevitable property of the follower role. For instance, when laying a table, the leader can choose to start with plates at any point of the table, which introduces a geometric constraint for the follower to put cutlery. Alternatively, imagine two people, a leader and a follower, loading a bike into a car rack; when the leader has grasped the bike, the follower has to grasp that same bike at the other end. Therefore, one can claim that this difference between the affordances of leaders versus followers is necessary to have an ecologically valid setup.

Our combination of dual eye tracking, multi-camera motion capture, and touch sensors enabled us to segment cooperative action into precise temporal intervals and to demonstrate not only the well-established precedence of gaze before action, but also striking role differences in how Leaders and Followers plan and monitor their behavior.

Clear differences also emerged between acting and observing. Leaders made more fixations when acting than when observing, a pattern not seen in the Follower. At one level, this reflects the task structure: Leaders were responsible for initiating each move and therefore had to invest more visual planning, whereas Followers only needed to identify what had to be undone. What is informative, however, is that the gaze data reveal how this division of labor translated into attentional priorities. Leaders devoted most of their fixations to planning their own actions, with comparatively little monitoring of the Follower, while Followers focused selectively on the Leader’s object choices, largely ignoring destinations. These asymmetries illustrate how role assignment shapes not only motor behavior but also the allocation of visual attention in joint action.

Further asymmetries between Leader and Follower were evident in fixation latencies. That Leaders look-ahead to both the object and the destination was, to some extent, imposed by the task structure: the Leader had to initiate each new sequence, whereas the Follower could only act in response. What is noteworthy, however, is how clearly this asymmetry manifested in the gaze data, revealing a consistent temporal gap between proactive planning in Leaders and reactive adjustment in Followers (see Figure 6A). This pattern resonates with broader accounts of proactive versus reactive control, and highlights how role assignment in joint action shapes the timing and content of visual planning.

Fixation duration also proved informative, echoing findings by van der Laan et al. (2015). Actors showed reliably longer fixations than observers, reflecting the greater attentional investment required for action planning. Importantly, the interaction between activity and event revealed a systematic shift in visual priorities: during action, participants fixated longer on the destination, whereas during observation, they focused more on the object to be moved. This dissociation demonstrates how role assignment shapes the functional allocation of gaze — either to guide one’s own unfolding movement or to track the co-player’s choice of object. The pattern aligns with previous work showing that in pick-and-place actions, people fixate on the object until it is lifted and then shift their gaze to the destination until release (Lavoie et al., 2018). Consistent with this, predictive gazes have also been documented in observers, who tend to look ahead to the actor’s goal rather than simply tracking the hand (Flanagan et al., 2013). Our study complements these findings by disentangling how such predictive gaze patterns differ between acting and observing in a cooperative, role-based task.

Leaders often produced alternating sequences of fixations between object and destination (Figure 10), a hallmark of planning-related search behavior. Look-ahead fixations of this type have been described before (Sullivan et al., 2021; Mennie et al., 2007; Pelz and Canosa, 2001). What our data add is evidence that such fixations frequently unfolded as extended back-and-forth sequences, linking objects and destinations in longer planning chains. This suggests that Leaders were not merely preparing the next immediate move but engaged in multi-step planning, a form of anticipatory gaze behavior not systematically documented in earlier work.

Our analyses further show that Leaders’ predictive eye movements extended beyond the next immediate action. As illustrated in Figure 6G, gaze peaks in intervals 4 and 5 revealed that even while waiting to act, Leaders frequently inspected potential objects the Follower might manipulate. This suggests that Leaders sometimes simulated not only their own upcoming moves but also the likely responses of their partner. Overrepresented sequences such as “own object–follower’s object–own object” or “own object–follower’s location–own object” point to a form of dual planning in which Leaders anticipated both their own and the Follower’s future actions. Such role-crossing predictions highlight the cognitive depth of coordination in this task.

Followers also displayed anticipatory gaze. Within ~400 ms after the Leader’s hand movement onset, they had already fixated the Leader’s intended target object (Figure 6). This rapid anticipation indicates that Followers did not simply trail the Leader’s hand but actively predicted the unfolding action. In this way, coordination in the task reflected a reciprocal prediction process, with Leaders sometimes anticipating the Follower’s responses and Followers anticipating the Leader’s choices.

Another observation concerns the “tails” following the main gaze peaks shown in Figure 6, which is noticeable for both acting Leaders and, even more, observing Followers. Many of these fixations were directed to locations where an object had just been removed, effectively gazes into the past. One might interpret these retrospective looks either as memory-related (O'regan, 1992; Johansson and Johansson, 2014; Foerster, 2019). In our study, for followers, remembering the pre-action state was essential to perform the undo operation. For leaders, such retrospective checks may have served to verify the configuration and to monitor whether the follower subsequently executed the correct undo action. However, this interpretation should be treated cautiously as we did not have any memory measurements, and hence, cannot rule out the possibility that these back gazes would be related to other cognitive processes.

It is essential to distinguish the current study from those that have examined gaze in cooperative settings involving reference acts (e.g., saying “look at the cup” or pointing; Gergle and Clark, 2011; Andrist et al., 2015). As the current study does not use verbal communication, gestures, or other referencing cues, the resulting joint action is qualitatively different. Therefore, in our discussion, we focused on studies with similar settings to ours.

The study has several limitations; first, when performing time-resolved analysis, we used the frequentist approach to test the observed differences, as time-resolved analysis was exploratory rather than confirmatory. Thus, the observations obtained therein shall be treated with care and be corroborated by confirmatory hypothesis testing.

Second, although in our experiment, we instructed participants not to communicate with each other, we did not implement any formal measure to rule out implicit non-verbal communication between our dyads (Canigueral and Hamilton, 2019; Cheng et al., 2023). Notably, in our experiments, players spent around 10% of the time with their eyes not directed toward objects or hands (i.e., on average 7.5%, for actor–leader, 8.7% for actor–follower, 11.1% for observer–leader, and 13.3% for observer–follower). However, one should consider that the game was cognitively demanding, and players appeared to focus strongly on the task. The short video clips of the game (see Supplementary Video “action-counteraction_experiment.mp4”) are representative of the timing of the actions, which follow each other quite rapidly. Since we did not register gazes at the face of partners (i.e., the partner’s face was not in the field of view of the eye-tracker), we cannot implement a video check in the current study to rule out implicit communications fully. However, future studies should implement such measures to rule out any implicit communication between dyads.

In sum, this study highlights how leader and follower roles elicit distinct decision-making and planning strategies in a continuous, naturalistic setting. Leaders displayed extended sequences of proactive fixations, sometimes spanning several steps and even anticipating potential moves of their partner. Followers, meanwhile, rapidly identified the leaders’ intended targets and combined predictive with retrospective gaze patterns to support the undo task. Both roles also exhibited looks to previously occupied locations, which could be related to further information collection and memory retrieval. Together, these findings reveal that joint action relies on a dynamic interplay of proactive planning, reciprocal prediction, and retrospective checking—processes that extend beyond the immediate next move, underscoring the complexity of everyday human coordination.

## Data Availability

The raw data supporting the conclusions of this article will be made available by the authors, without undue reservation.

## Footnotes

1 http://www.R-project.org/

2 https://mc-stan.org/
