# Supplementary material for: Analyzing gaze and hand movement patterns in leader-follower interactions during a time-continuous cooperative manipulation task
Source: Front Psychol. 2026 Jan 6;16:1699261. doi: 10.3389/fpsyg.2025.1699261 (PMC12815842; doi:10.3389/fpsyg.2025.1699261)
Supplement: Supplementary file 5 [file Supplementary_method_results.pdf]

Supplement for

Analyzing Gaze and Hand Movement Patterns in Leader-Follower Interactions During a Time-Continuous Cooperative Manipulation Task

Minghao Cheng[1*], Anoushiravan Zahedi[3,4*], Ricarda I. Schubotz[3,4] Florentin Wörgötter[1], and Minija Tamosiunaite[1,2*]

[1]Inst. Physics 3, Computational Neuroscience, Georg-August University Göttingen, Germany

[2]Faculty of Informatics, Vytautas Magnus University, Kaunas, Lithuania

[3]Department of Psychology, University of Münster, Münster, Germany

[4]Otto-Creutzfeldt-Center of Behavioral and Cognitive Neuroscience, University of Münster, Münster, Germany

This supplement provides additional information concerning the computer vision algorithms used to record experimental data as well as results and diagrams for different additional aspects including the statistical-modeling based analysis.

Computer vision - Ray tracing

The algorithm consists of two main parts: ray casting and tracing. By combining the head pose data and the eye tracker – camera setup transformation matrix, the fixation point is converted into a ray in the 3D space, where its origin is the origin of the scene camera, and the direction vector is calculated by connecting the origin and the fixation point. Mathematically, a ray r, defined as $\vec{O}+\vec{D}\cdot t$, $\vec{O}$ represents the origin of the ray, and it is the origin of the scene camera of the eye tracker. $\vec{D}$ is the direction vector, which connects the origin and the gaze fixation point on the scene camera's image plane, t is a positive scalar that extends the ray from its origin towards infinity. The tracing algorithm is essentially a collision detection between the casted ray and the 3D AABBs, and it operates by treating each axis individually and then combining their results. In each dimension, a 3D AABB is defined by two lines: one with minimum coordinates and one with maximum coordinates. The intersection area of these lines in 3D space forms the AABB. Each line $l_{n,m}$can be expressed as $Y = B_{n,m}$, where n is the index of the dimension and m can be either "*max*" or "*min*". The solution of the following equation indicates where does ray r hits one of these lines: $O_n + t_{n,m} \cdot D_n = B_{n,m}$. The result $t_{n,m}$ give the ranges where the ray r hits the boundary lines of the AABB in each dimension by illustrating the extent of the direction vector $\vec{D}$. In the case where r hits the AABB, there will be an overlap range across the ranges of all dimensions, that is: $[t_{x,min}, t_{x,max}] \cap [t_{y,min}, t_{y,max}] \cap [t_{z,min}, t_{z,max}] \neq \emptyset$ Moreover,

there will be a pair of $t_{n,m}$ that define the range of the ray which penetrates through the AABB, t'_{min} and t'_{max} can be used to annotate them. Then a point $\vec{P} = \vec{O} + \vec{D} \cdot t'_{min}$ is the point where the ray hits the surface of the AABB in the first place. In contrast, when the ray r does not hit the AABB, there is no overlap of $t_{n,m}$ across all dimensions. By executing the ray tracing algorithm with each 3D AABB, the location data can be extracted. If the ray intersects both an object and a hand, the hand is prioritized as the output.

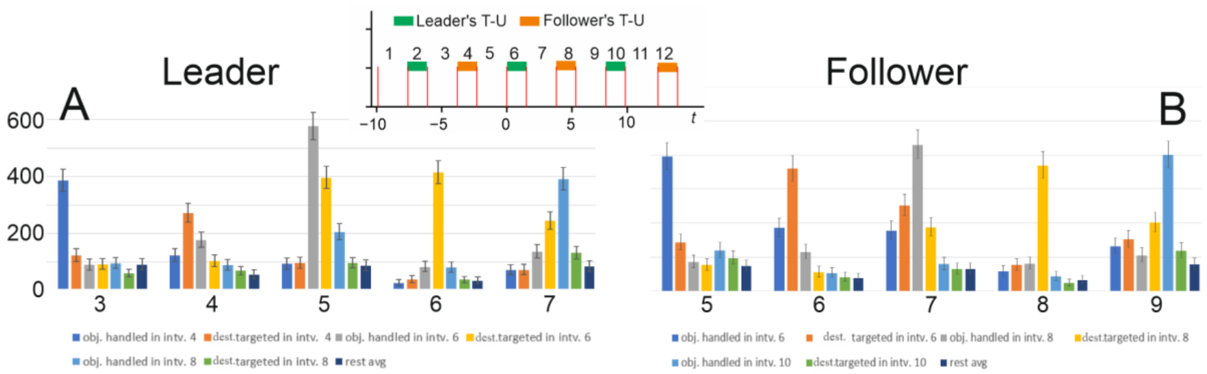

Figure 1) Objects and destinations per interval. Intervals (abscissa) are numbered as shown in the inset above.

Fixation distribution in consecutive T-U and U-T intervals

In Figure 1 we analyze sequences of time intervals (T-U and U-T), considering at which objects/destinations Leader and Follower have looked in those different intervals. In a nutshell: This diagram shows, quite expectedly, that the different “aspects of interest” are indeed looked at one after the other.

A total of 500 sample-intervals has been analyzed to obtain a histogram for each interval. Thus, histograms show the number of fixations per 500 intervals. Error bars represent 95% confidence intervals calculated for Poisson distributions.

The Leader has finished their action in interval 2 and begins considering their next action but before they look quite often at what the Follower does. Hence, fixations at the Follower’s object (blue bar, left) are common in interval 3 (compare also to Figure 6 G in the main text). Note that these fixations occur before the Follower has actually touched their object, which happens only at the start of 4. During this time the Leader shifts gaze to the targeted destination of the Follower (orange bar). However, the Leader now also begins to consider, which object to manipulate next (gray bar). As expected, this becomes prevalent in interval 5, but here the Leader also starts to search for the placing destination (yellow bar). During execution of the Leader’s action (intv. 6) fixation on the destination is dominant. Note that interval 7 is equivalent to 3 but not identical. This is due to the fact that intervals follow each other to define an episode and the later coming intervals are conditional on the earlier ones, leading to similar but not identical results for equivalent intervals. Hence for 7 the light blue bar in 7 corresponds to the dark blue one in 3 (same for green and orange). Indeed, in interval 7 the fixations at the next object of the Follower again begin to dominate (as in 3), but here the Leader still keeps looking at their chosen destination (yellow), too.

For the Follower a quite similar pattern emerges. However, in interval 6 and 7 the dark blue and orange bar are larger for the Follower than the equivalent counterparts for the Leader (which are found in intervals 4 and 5). For both of them, these two bars represent “what the other does” and, evidently, this is more relevant for the Follower than for the Leader.

Additional material about the statistical modelling-based analysis

Here we provide additional material concerning the statistical analyzes of the fixation patterns (length, number, latency). In all three cases the statistical model converged correctly without any divergent transitions (all $Rhat = 1$) as described in the main text. Further, bulk and tail effective sample sizes for main effects and interactions were above 4000, showing that model predictions were reliable. Furthermore, the model accurately captured the observed data distribution.

Fixation counts

In Table 1, we provide details on hypothesis testing for fixation counts, i.e., the number of fixations as modeled by Equation 2, with main factors of activity (Actor vs. Observer), role (Leader vs. Follower), and event (Touching vs. Untouching events). For details on the model, please refer to the main text.

Table 1. Hierarchical Bayesian generalized linear modelling results for the fixation count (Eq. 2 in the main text)

**Hypothesis**	***Estimate (SE)***	***95% CI***	***BF_{01}***	***Post Prob***
Activity = 0	0.11 ± 0.04	[0.02, 0.19]	1.89	0.65
Role = 0	0.03 ± 0.04	[-0.05, 0.11]	38.25	0.97
Event = 0	-0.05 ± 0.03	[-0.12, 0.02]	23.67	0.96
**Time Window = 0**	0.19 ± 0.01	[0.17, 0.21]	**0.00**	**0.00**
**Activity * Role = 0**	0.21 ± 0.05	[0.10, 0.31]	**0.01**	**0.01**
Activity * Event = 0	0.04 ± 0.05	[-0.06, 0.13]	**30.58**	**0.97**
**Role * Event = 0**	0.19 ± 0.05	[0.10, 0.28]	**0.00**	**0.00**
**Activity * Role * Event = 0**	-0.23 ± 0.06	[-0.36, -0.11]	**0.06**	**0.05**

Note: Significant alternative hypotheses are indicated in bold.

CI: Credential interval.

BF_{01}: Bayes Factor comparing the Null hypothesis to the alternative hypothesis. All hypotheses were tested using the brms package (Bürkner 2017). Based on the suggestion of van Doorn et al. (2021), $Bayes\ factors(BF) > 3$ were considered as significant evidence for the null hypothesis and $BF < 1/3$ for the alternative hypothesis.

Fixation duration

In Table 2, we provide details on hypothesis testing for fixation duration, i.e., the cumulative duration of fixations, as modeled via Equation 3. For details on the models, please refer to the main text.

Table 2. Hierarchical Bayesian generalized linear modelling results for the fixation duration (Eq. 3 in the main text)

**Hypothesis**	***Estimate (SE)***	***95% CI***	***BF_{01}***	***Post Prob***
**Activity = 0**	0.18 ± 0.03	[0.12, 0.25]	**0.00**	**0.00**
Role = 0	-0.08 ± 0.04	[-0.15, -0.01]	4.77	0.83
Event = 0	0.05 ± 0.03	[-0.01, 0.11]	**19.66**	**0.95**
**Time Window = 0**	0.14 ± 0.01	[0.12, 0.15]	**0.00**	**0.00**
**Activity * Role = 0**	0.20 ± 0.05	[0.10, 0.29]	**0.01**	**0.01**
**Activity * Event = 0**	-0.19 ± 0.04	[-0.27, -0.11]	**0.00**	**0.00**
Role * Event = 0	0.06 ± 0.04	[-0.02, 0.14]	**16.00**	0.94
Activity * Role * Event = 0	-0.01 ± 0.06	[-0.12, 0.10]	**34.61**	**0.97**

Note: Significant alternative hypotheses are indicated in bold.

CI: Credential interval.

BF_{01}: Bayes Factor comparing the Null hypothesis to the alternative hypothesis. All hypotheses were tested using the brms package (Bürkner 2017). Based on the suggestion of van Doorn et al. (2021), $Bayes\ factors(BF) > 3$ were considered as significant evidence for the null hypothesis and $BF < 1/3$ for the alternative hypothesis.

Fixation latency

In the same way as for the other two aspects above, in Table 3, we provide details on hypothesis testing on fixation latency, i.e., how far in advance of a predefined event a fixation is elicited as modeled via Equation 4. For details of the model, please refer to the main text.

Table 3. Hierarchical Bayesian generalized linear modelling results for the fixation latency (Eq. 4 in the main text)

**Hypothesis**	**Estimate (SE)**	**95% CI**	**Evid Ratio**	**Post Prob**
Activity = 0	-0.07 ± 0.03	[-0.14, 0.00]	**3.12**	0.76
Role = 0	0.15 ± 0.03	[0.09, 0.22]	**0.00**	**0.00**
Event = 0	-0.12 ± 0.03	[-0.18, -0.05]	**0.02**	**0.02**
**Time Window = 0**	0.29 ± 0.01	[0.28, 0.31]	**0.00**	**0.00**
Activity * Role = 0	-0.04 ± 0.04	[-0.12, 0.05]	**14.55**	0.94
**Activity * Event = 0**	0.14 ± 0.04	[0.06, 0.22]	**0.12**	0.11
**Role * Event = 0**	0.12 ± 0.04	[0.04, 0.20]	**0.35**	0.26
Activity * Role * Event = 0	-0.13 ± 0.06	[-0.24, -0.02]	1.34	0.57

Note: Significant alternative hypotheses are indicated in bold.

CI: Credential interval.

BF_{01}: Bayes Factor comparing the Null hypothesis to the alternative hypothesis. All hypotheses were tested using the hypothesis package from brms (Bürkner 2017). Based on the suggestion of van Doorn et al. (2021), $Bayes\ factors(BF) > 3$ were considered as significant evidence for the null hypothesis and $BF < 1/3$ for the alternative hypothesis.

References

Bürkner, P.-C. (2017). brms: An R Package for Bayesian Multilevel Models Using Stan. Journal of Statistical Software, 80(1).

van Doorn, J., van den Bergh, D., Bohm, U., Dablander, F., Derks, K., Draws, T., . . . Wagenmakers, E. J. (2021). The JASP guidelines for conducting and reporting a Bayesian analysis. Psychon Bull Rev, 28(3), 813-826.
